# Nuclear exchange generates population diversity in the wheat leaf rust pathogen *Puccinia triticina*

**DOI:** 10.1038/s41564-023-01494-9

**Published:** 2023-10-26

**Authors:** Jana Sperschneider, Tim Hewitt, David C. Lewis, Sambasivam Periyannan, Andrew W. Milgate, Lee T. Hickey, Rohit Mago, Peter N. Dodds, Melania Figueroa

**Affiliations:** 1 Black Mountain Science and Innovation Park, CSIRO Agriculture and Food, GPO, Canberra, Australian Capital Territory Australia; 2 NSW Department of Primary Industries, Wagga Wagga Agricultural Institute, Wagga Wagga, New South Wales Australia; 3 Queensland Alliance for Agriculture and Food Innovation, The University of Queensland, St Lucia, Queensland Australia; 4 Present Address: School of Agriculture and Environmental Science, Centre for Crop Health, The University of Southern Queensland, Toowoomba, Queensland Australia

**Keywords:** Pathogens, Fungi

## Abstract

In clonally reproducing dikaryotic rust fungi, non-sexual processes such as somatic nuclear exchange are postulated to play a role in diversity but have been difficult to detect due to the lack of genome resolution between the two haploid nuclei. We examined three nuclear-phased genome assemblies of *Puccinia triticina*, which causes wheat leaf rust disease. We found that the most recently emerged Australian lineage was derived by nuclear exchange between two pre-existing lineages, which originated in Europe and North America. Haplotype-specific phylogenetic analysis reveals that repeated somatic exchange events have shuffled haploid nuclei between long-term clonal lineages, leading to a global *P. triticina* population representing different combinations of a limited number of haploid genomes. Thus, nuclear exchange seems to be the predominant mechanism generating diversity and the emergence of new strains in this otherwise clonal pathogen. Such genomics-accelerated surveillance of pathogen evolution paves the way for more accurate global disease monitoring.

## Abstract (web summary)

Sperschneider et al. generate nuclear-assigned genome assemblies for several isolates of the wheat leaf rust pathogen, *Puccinia triticina*, and show that repeated nuclei shuffling between clonal lineages has created global populations.

## Main

Rust fungi (order Pucciniales) cause diseases on important agricultural crops and threaten food production and ecosystems. For *Puccinia* species, the asexual (uredinial) phase of their life cycle infects cereal hosts, while the sexual phase occurs on different host plants. Thus, rust population dynamics varies from highly sexual to exclusively clonal depending on the presence and abundance of the alternate host^1^. For instance, *Puccinia coronata* f. sp. *avenae* (*Pca*) populations causing oat crown rust disease are highly genetically diverse in North America where the sexual host buckthorn is prevalent^2,3^. In contrast, *Puccinia graminis* f. sp. *tritici* (*Pgt*) populations that cause wheat stem rust disease are clonal in most parts of the world, but local sexual populations occur where the alternate host barberry (*Berberis* spp.) is present^4,5^. Wheat leaf rust disease caused by *Puccinia triticina* (*Pt*) results in substantial crop losses around the world^6,7^, with its sexual host, *Thalictrum* spp., being scarce in North America and Europe and absent in Australia^8^. Genetic analyses indicate that global populations of *Pt* consist of relatively few major clonal lineages, with high levels of heterozygosity and linkage disequilibrium and low diversity within lineages, consistent with a lack of sexual recombination^9–12^. In Australia, five clonal lineages of *Pt* have been described, apparently derived from exotic incursions^13,14^.

In the absence of sexual reproduction, evolution of rust fungi is limited to mutation and somatic exchange events^1^. Early laboratory studies showed that somatic genetic exchange of virulence genes can occur between two rust isolates infecting the same plant^15–19^, with some evidence of somatic hybridization occurring in the field for *Pgt* and *Pt* based on limited molecular markers^20,21^. Models proposed for somatic hybridization ranged from simple exchange of nuclei of opposite mating type to parasexual recombination, but the only genetically controlled analysis to discriminate between these possibilities was conducted in flax rust (*Melampsora lini*)^17^. In this case, no recombination occurred between several avirulence loci with known nuclear genotypes and clear +/− compatibility groups were detected, but this was not confirmed in other rust species. However, recent analysis of fully nuclear-phased genome assemblies clearly demonstrated that somatic exchanges of whole nuclei have contributed to genetic diversity in *Pgt*^22^. The Ug99 lineage of *Pgt*, which emerged in 1998, shares a single nucleus-specific haplotype with the much older South African *Pgt*21 lineage, while three other globally dispersed isolates share common nuclear genotypes with either *Pgt*21-0 or Ug99. Genome admixture analyses suggested that another five *Pgt* lineages may be derived by somatic exchange^23^. We previously generated a fully nuclear-phased chromosome genome assembly for an Australian isolate of *Pt* (*Pt*76) (ref. ^24^) and here we extend this to two additional isolates and use these references to compare haplotype diversity across a large set of sequenced *Pt* isolates from around the world. This reveals evidence of extensive nuclear exchange events underlying the origin of major clonal lineages, indicating a very substantial contribution of somatic hybridization to population dynamics.

## Results

### Seven recent Australian *Pt* isolates form three lineages

Six Australian isolates of *Pt* collected in 2019 and 2020 exhibited four virulence pathotypes (Supplementary Table 1). The 19QLD08 isolate shared the same pathotype as *Pt*76 (=19ACT06) (ref. ^24^) but with virulence for *Lr20*. Both of these are identical to pathotypes found in a lineage derived from pathotype 76-3,5,9,10 + Lr37, first detected in Australia in 2005 (refs. ^13,14^). The 20QLD87 isolate has the same pathotype as a lineage (104-1,3,4,6,7,8,9,10,12 + Lr37) first detected in 2014 as an apparent exotic incursion into Australia via New Zealand^25^. The 20ACT90 isolate shares a pathotype with the currently predominant lineage in Australia (104-1,3,4,5,7,9,10,12 + Lr37), which was first detected in 2016 (ref. ^26^), while 19NSW04, 19ACT07 and 20QLD91 share the same pathotype but with additional virulence for *Lr27*/*Lr31*.

**Fig. 1:**
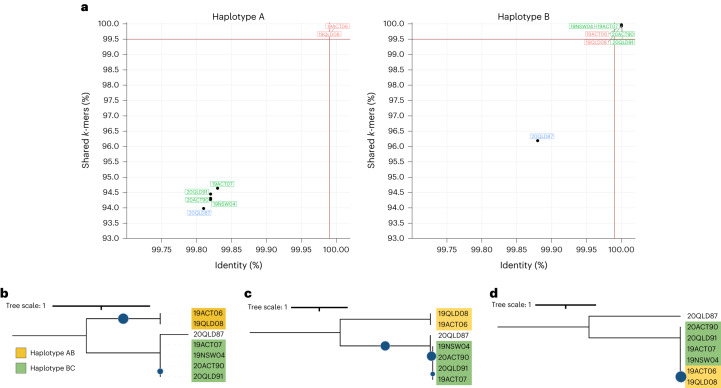
Three distinct lineages and four haplotypes are present in a collection of seven Australian *Pt* isolates. **a**, *k*-mer genome containment scores of Illumina sequencing reads against the *Pt*76 (19ACT06) haplotypes. Identity is the percentage of bases that are shared between the genome and the sequencing reads. Shared *k-*mers is the percentage of *k*-mers shared between the genome and the sequencing reads. Two red lines indicate thresholds above which we consider a haplotype genome to be fully contained in the sequencing reads of an isolate (identity ≥99.99%, shared *k*-mers ≥99.5%). The A haplotype is fully contained in the sequencing reads of two isolates (19ACT06, 19QLD08) and the B haplotype is fully contained in the sequencing reads of 6 isolates (19ACT06, 19QLD08, 19ACT07, 19NSW04, 20QLD91, 20ACT90). The 20QLD87 isolate contains neither A nor B haplotypes. **b**, The phylogenetic tree against the combined haplotypes 19ACT06 A and B indicate three lineages. **c**, The phylogenetic tree against the 19ACT06 haplotype A shows that two isolates share the A haplotype. **d**, The phylogenetic tree against the 19ACT06 haplotype B shows that six isolates share the B haplotype. Bootstrap values of over 80% are indicated with blue circles.

We generated Illumina genomic sequences from these isolates and used a *k*-mer containment analysis to compare their nuclear haplotype similarity to the 19ACT06 reference genome^24^. Figure 1a shows the proportion of genome *k*-mers represented as identical sequences in the Illumina data (shared *k*-mers) against the overall sequence similarity of *k*-mers to the Illumina data (*k*-mer identity) for the 19ACT06 A and B haplotypes. Illumina reads from 19ACT06 and 19QLD08 fully contained the *k*-mers (99.9% shared *k*-mers and 100.00% overall *k*-mer identity) from both nuclear haplotypes, confirming that these isolates are the same clonal lineage. However, while the B haplotype is also fully contained in the Illumina reads of 20ACT90, 20QLD91, 19ACT07 and 19NSW04, the A haplotype is not (only ~94% shared *k*-mers and 99.80% *k*-mer identity), suggesting that these isolates share the B nuclear haplotype with another divergent haplotype (C). Neither the A nor the B haplotypes are fully contained in the Illumina reads of 20QLD87 (94–96% shared *k*-mers, 99.80–99.87% *k*-mer identity), suggesting a different unknown genomic composition. These relationships were confirmed by haplotype-specific phylogenetic trees based on single nucleotide polymorphisms (SNPs). In trees based on the full diploid genome (Fig. 1b) or the A haplotype (Fig. 1c), these isolates fell into three distinct lineages designated AU1 (19ACT06 and 19QLD08), AU2 (20ACT90, 19NSW04, 19ACT07, 20QLD91) and AU3 (20QLD87). However, the AU1 and AU2 isolates grouped together in a single closely related clade in a tree based on only the B genome SNPs (Fig. 1d).

### Nuclear-phased genomes for members of the three lineages

**Extended Data Fig. 1:**
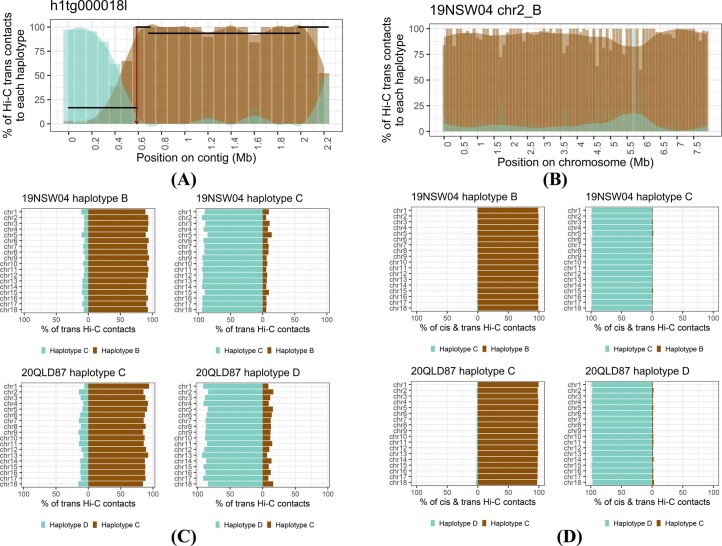
Percentage of Hi-C contacts that link within and between nuclear-separated haplotypes. **(a)** The % of Hi-C *trans* contacts that link to the haplotypes C (turquoise) and B (brown) in each 100kbp bin (with an associated smoothing lines) are shown for hifiasm contig h1tg000018l, which contains a phase switch site (indicated by red arrow). HiCanu haplotig alignment positions (black segments) are shown at the y-coordinate that corresponds to their Hi-C contacts to haplotype C. **(b)** The % of Hi-C *trans* contacts that link to each haplotype for the fully phased 19NSW04 chromosome 2B. **(c** and **d)** Graphs showing the % of Hi-C *trans* contacts (left panel) or *cis* and *trans* contacts (right panel) that link to each haplotype for the 20QLD87 and 19NSW04 chromosome assemblies.

To further analyse haplotype similarity in these isolates, we generated nuclear-phased genome assemblies with PacBio HiFi and Hi-C data for 19NSW04 (AU2) and 20QLD87 (AU3) using hifiasm with Hi-C integration. Each haplotype assembly was 123–129 Mb in size, highly contiguous (L50 > 6 Mb) and with BUSCO completeness of over 95% (<5% duplicated) (Supplementary Table 2). The NuclearPhaser pipeline^24^ showed that the haplotype assemblies were nearly perfectly nuclear-assigned, with only two contigs larger than 150 kb (1.2 Mb total) assigned to the incorrect phase in 19NSW04 and a single mis-assigned contig (2.2 Mb) in 20QLD87 (Supplementary Fig. 1), with potential phase switches detected in only three contigs from 20QLD87 and none from 19NSW04. Previously we observed that phase switches occurred at haplotig boundaries^22,27^ in assemblies generated by Canu^28,29^, which breaks contigs at points of phase ambiguity. However, this was not the case in all of these contigs generated by hifiasm which aims to reconstruct both homologous haplotypes with high contiguity^30^. We therefore re-assembled the 20QLD87 HiFi reads with HiCanu and found that the predicted phase-switch regions in all three hifiasm contigs correspond to boundaries between HiCanu haplotigs which also switch phase at that site (for example, h1tg000018l; Extended Data Fig. 1a), and these coordinates were used as breakpoints to correct the phase switches.

Contig scaffolding resulted in 18 chromosomes for each nuclear haplotype of 19NSW04 and 20QLD87 (Supplementary Fig. 2a). Over 99% of *cis* and *trans* Hi-C links occur within a nucleus, supporting the correct phasing of homologous chromosomes, with over 90% of *trans* Hi-C links occurring within a nucleus, as previously observed for dikaryons^22,24,27,31^, and supporting the correct nuclear assignment of chromosome pairs (Extended Data Fig. 1b–d). The four chromosome haplotype assemblies range from 121.8 Mb to 123.8 Mb in length (Supplementary Table 3), similar to isolate 19ACT06 (121.6 Mb and 123.9 Mb) (ref. ^24^). Additional unplaced contigs are small (L50 of 76.2 Kb or 38.7 Kb), containing mainly repetitive sequences, especially rRNAs, with few genes (Supplementary Table 3). We annotated genes in 19NSW04 and 20QLD87 and re-annotated 19ACT06 using a pipeline optimized for effector annotation, which identified about 18,000 genes in each haploid genotype (Supplementary Table 3). This represented an increased number of genes in 19ACT06 from the previously reported 29,052 (ref. ^24^) to 36,343 (haplotype A: 17,958 genes; haplotype B: 17,813 genes), including an increase of 49.1% in annotated genes encoding secreted proteins compared with only 18.6% more genes encoding non-secreted proteins.

### The three lineages share nuclear haplotypes

**Fig. 2:**
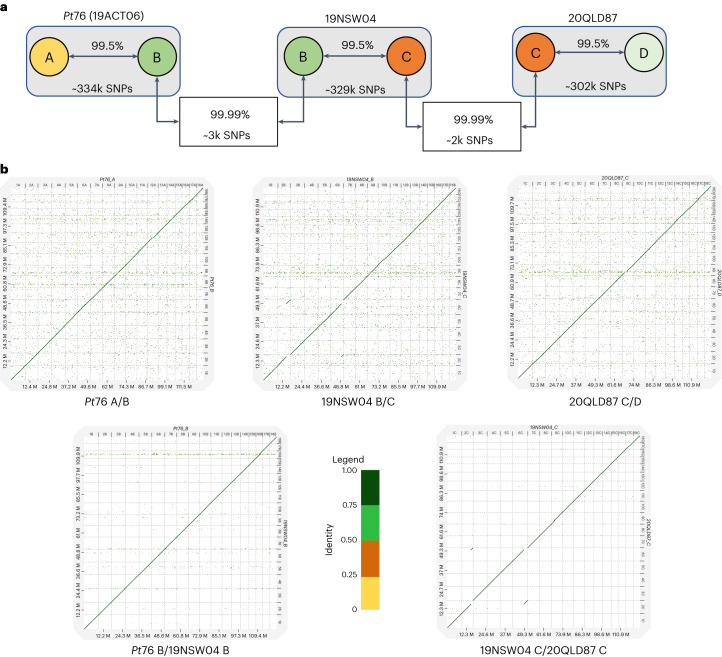
*Pt76* (19ACT06) and 19NSW04 share a near-identical copy of haplotype B, and 19NSW04 and 20QLD87 share a near-identical copy of haplotype C. **a**, Diagram showing the average identity of genomic alignments and total number of SNPs among haplotypes of 19ACT06, 19NSW04 and 20QLD87. **b**, Dot plot of genomic alignments showing a single translocation in the 19NSW04 C haplotype.

Genome sequence alignment showed that within each isolate, the two separate haplotypes have average sequence identity of 99.50% (divergence 0.50%), with ~303,000 to 334,000 distinguishing SNPs (Fig. 2a and Supplementary Table 4). However, one of the 19NSW04 haplotypes shares remarkably high sequence similarity with the 19ACT06 B haplotype with only 2,966 SNPs and average sequence alignment identity of 99.99% (divergence 0.01%), while the other 19NSW04 haplotype shares similarly high sequence identity (99.99%, 2,182 SNPs) with one of the 20QLD87 haplotypes. Thus, we assigned the 19NSW04 haplotypes as B and C and the 20QLD87 haplotypes as C and D (Supplementary Table 2 and Fig. 2a). The 19NSW04 C haplotype contains a translocation between chromosomes 2 and 6, which is not present in any of the other haplotypes, including the 20QLD87 C haplotype (Fig. 2b), and this translocation is supported by Hi-C contact maps and HiFi read coverage across the breakpoints (Supplementary Fig. 2a). The shared B and C haplotypes suggest that these isolates are related by nuclear exchange, with the simplest scenario that the AU2 lineage (BC haplotype) arose by somatic hybridization between isolates of the AU1 (AB) and AU3 (CD) lineages given its most recent detection in Australia^13,14,25,26^.

**Fig. 3:**
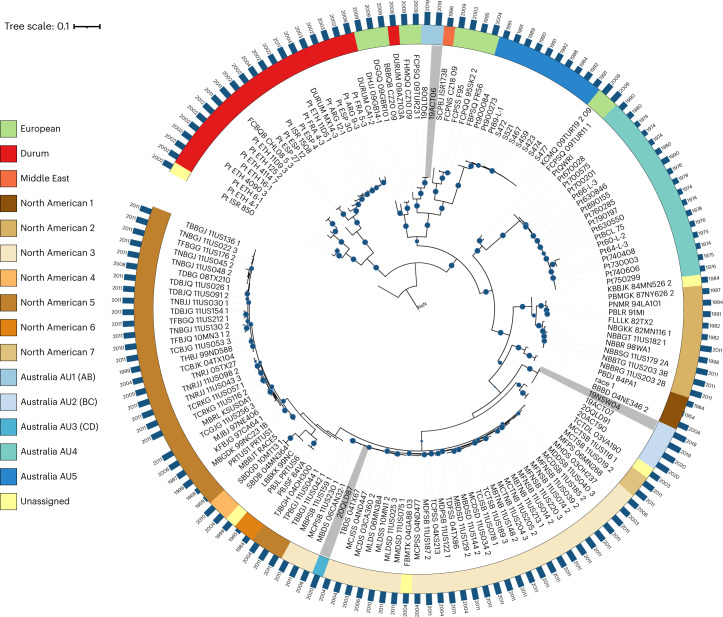
Phylogenetic tree with the diploid genome of 19NSW04 (BC) as the reference. A maximum-likelihood tree was generated from 175,974 bi-allelic SNPs. Isolates from the five Australian *Pt* clades (AU1–AU5), seven North American clades (NA1–NA7) or Europe are indicated by colour according to the legend inset. Five isolates are unassigned to clades, that is, Pt_ISR_850 (*Aegilops speltoides*, outgroup), 03VA190 (unassigned in ref. ^34^) and three others which are in disagreement with the clade assignment given in ref. ^34^. *Pt* isolates with fully phased haplotype genome references are highlighted in grey. Bootstrap values over 80% are indicated with blue circles. The year of collection for each isolate is shown next to the blue bars which indicate time passed since 1950.

**Fig. 4:**
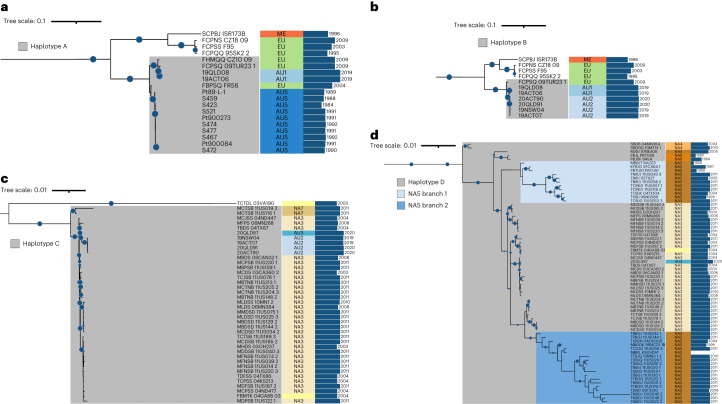
Pruned phylogenetic trees of global *Pt* isolates against the individual A, B, C and D haplotypes. **a**–**d**, Phylogenetic trees were constructed on the basis of SNPs called against the single haplotypes (Extended Data Figs. 4–7) and sub-branches of the trees containing isolates with the relevant haplotype reference are displayed. **a**, 19ACT06 haplotype A. **b**, 19NSW04 haplotype B. **c**, 20QLD87 haplotype C. **d**, 20QLD87 haplotype D. Bootstrap values over 80% are indicated with blue circles. Clades are indicated next to the name of each isolate (ME, Middle East; EU, European; AU1–AU5, Australian 1–5; NA1–7, North American 1–7). The year of collection for each isolate is shown next to the blue bars.

**Fig. 5:**
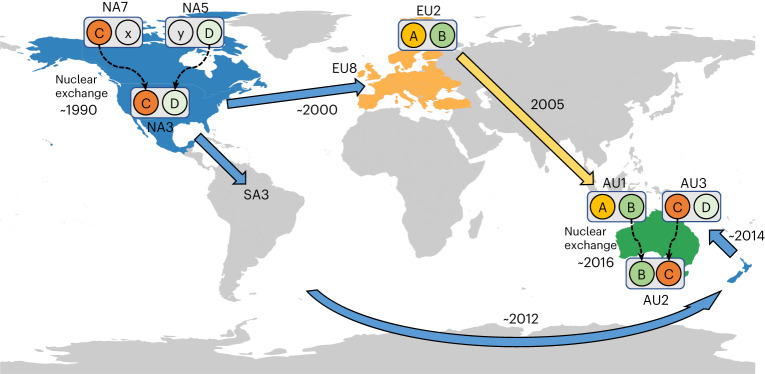
Nuclear exchange events have shaped global *Pt* lineages. The North American 3 (NA3) lineage most probably arose from somatic hybridization involving an isolate of the North American 5 (NA5) group which donated the D genome. The North American 7 (NA7) clonal group contains the C haplotype and could represent the parental lineage donating this haplotype to NA3. The NA3 lineage subsequently spread to other parts of the world, including Australia. The Australian 2 lineage (AU2, BC nuclear genotype) probably arose from somatic hybridization of the AB lineage (European 2, EU2) with the CD (Australian 3, AU3) lineage.

Over 80% of SNPs distinguishing the six complete haplotype assemblies (including between the two copies of B and C haplotypes) occur in repetitive sequences, with only ~10% in coding regions, of which ~59% are non-synonymous (Supplementary Tables 5 and 6, and Figs. 3–7). This corresponds to coding differences in genes encoding ~7,600 proteins (~15.5% secreted) between distinct haplotypes, and 122 (21 secreted) and 60 (5 secreted) proteins between the two copies of the B and C haplotype, respectively (Supplementary Data 1). As over 70% of these proteins lack functional annotation, further work is required to assess the role of this variation in *Pt* evolution.

### Clonal lineages with the AB and CD haplotypes occur globally

To investigate the origin of the Australian lineages, we used previously available whole-genome sequencing data from an additional 27 isolates from Australia and New Zealand^32,33^ and 120 worldwide isolates mostly from North America and Europe^34^ (Supplementary Data 1). A phylogeny derived from SNPs called against the 19NSW04 diploid genome (Fig. 3) shows very similar topology to a previously reported phylogeny for the 120 global isolates^34^. This largely confirmed the placement of North American isolates into six clades (NA1–6), except for five isolates originally classified in clades NA1 (99NC; 7 o’clock), NA2 (04GA88-03; 2 o’clock), NA3 (11US116-1 and 11US019-2; both 4 o’clock) and NA5 (84MN526-2; 3 o’clock). In the previous analysis^34^, these isolates were basal to and significantly diverged from these clusters, consistent with belonging to distinct lineages. We classified the 11US116-1 and 11US019-2 isolates as a separate clade NA7, since results below indicate that they contain a novel haplotype combination relevant to the evolution of the North American population. The Australian isolate 20QLD87 (AU3; CD haplotype) was placed within the NA3 group, indicating that it represents a clonal lineage that arrived in Australia as a result of intercontinental migration. The AU1 (AB) lineage closely groups with a Turkish isolate collected in 2009 (09TUR23-1), previously placed in the European group EU2 (ref. ^11^), suggesting a European origin of this lineage. The AU2 group (BC) did not cluster with other global isolates, consistent with an origin by hybridization in Australia. The older Australian isolates cluster in two clonal groups separate from the recent isolates; AU4 containing isolates collected between 1974 and 1990 (ref. ^33^); and AU5 containing isolates collected between 1984 and 1992 and representing a clonal lineage derived from pathotype 104-1,2,3,(6),(7),11 first detected as an exotic incursion in 1984 (refs. ^32,33^). The AU5 group is closely related to a French isolate (FR56) collected in 2004 and part of European clade EU7 (ref. ^11^).

### Clonal lineages share haplotypes in distinct combinations

We also constructed phylogenies using SNPs from the individual A, B, C and D haplotypes to identify lineages sharing these haplotypes (Fig. 4 and Extended Data Figs. 4–7). In an A haplotype phylogenetic tree (Fig. 4a and Extended Data Fig. 4), the AU1 isolates (AB) again form a clonal clade with the Turkish isolate (09TUR23-1, EU2), but also with an isolate collected in 2009 from Czech-Slovakia (CZ10-09, EU5), as well as with the AU5 group and the closely related FR56 isolate (EU7 group), suggesting that these groups all share a nucleus with very high similarity to the A haplotype of 19ACT06. In a B haplotype phylogenetic tree (Fig. 4b and Extended Data Fig. 5), the AU1 (AB) and AU2 (BC) groups form a clonal clade with isolate 09TUR23-1, again confirming that this EU2 isolate contains both the A and B haplotypes. In a C haplotype phylogenetic tree (Fig. 4c and Extended Data Fig. 6), the AU2 (BC) and 20QLD87 (CD) isolates form a clonal group with isolates from the North American clade 3 (NA3), again confirming their shared C haplotype. The C haplotypes of the AU2 isolates are most closely related to 20QLD87, which is consistent with 20QLD87 representing the parental lineage that donated the C nucleus to this hybrid lineage. Likewise, 20QLD87 (CD) again formed a clonal group including the NA3 isolates in a D haplotype phylogenetic tree (Fig. 4d and Extended Data Fig. 7). However, this group also included isolates from the North American clades NA4, NA5 and NA6, suggesting that they all share a common D haplotype. The NA3 group (CD) branches from within the NA5 group, indicating that the D genome in NA3 is probably derived from a parental isolate from NA5. The NA4 and NA6 groups branch from older nodes in this clade, indicating that their D genomes diverged earlier. In addition, three other North American isolates (99NC, 03VA190, 84MN526_2) that form singleton branches in the other phylogenetic trees were closely related and basal to this D genome-containing group, suggesting that they may contain versions of the D haplotype with even older divergence times (Extended Data Fig. 7). Notably, the two isolates in group NA7 (11US116-1 and 11US019-2) cluster with the 20QLD87 and NA3 isolates in the C haplotype phylogenetic tree only, suggesting that they share the C haplotype (Fig. 4c,d). The basal position to the NA3 clade in this tree with strong bootstrap support is consistent with these isolates representing the other parental lineage donating the C nuclear haplotype to the NA3 (CD) hybrid. Close examination of the D genome phylogenetic tree indicates that the NA5 group is divided into two separate branches with strong bootstrap support (Fig. 4d). Branch 1 is ancestral to NA3 consistent with being the D haplotype donor, while branch 2 diverged more recently from within the NA3 group. This suggests that a subsequent nuclear exchange event may have occurred in which the D genome of an NA3 isolate was swapped back into an NA5 isolate to recreate a similar haplotype combination but with a different evolutionary history for the D haplotype. A *k-*mer containment analysis (Table 1, Extended Data Fig. 8 and Supplementary Data 1) confirmed these haplotype relationships, with Illumina reads from isolates postulated to contain shared haplotypes showing >99.5 shared *k*-mers and >99.99% *k*-mer identity for those haplotypes. Thus, numerous clonal lineages share these four nuclear haplotypes in various combinations, suggesting that somatic nuclear exchange is common in *Pt* populations.

**Table 1:** *k*-mer genome containment scores against sequencing reads of various *Pt* isolates and clades

*Pt* isolate/clades	*k*-mer identity (%) / shared *k-*mers (%)				
	19ACT06 haplotype A	19NSW04 haplotype B	19NSW04 haplotype C	20QLD87 haplotype D	Haplotypes present
AU1	**100 / 99.9**	**100 / 99.9**	99.82 / 94.4	99.89 / 96.5	A,B
AU2	99.82 / 94.4	**100 / 100**	**100 / 100**	99.91 / 97.1	B,C
20QLD87 (AU3)	99.81 / 94.0	99.88 / 96.2	**100 / 100**	**100 / 100**	C,D
AU5	**99.99 / 99.7**	99.87 / 96.0	99.87 / 95.9	99.88 / 96.1	A
AU4	99.87 / 95.8	99.87 / 95.9	99.87 / 95.8	99.90 / 96.9	Unidentified
09TUR23_1	**99.99 / 99.8**	**99.99 / 99.9**	99.90 / 96.9	99.94 / 98.1	A,B
FR56	**99.99 / 99.7**	99.93 / 97.8	99.90 / 97.0	99.92 / 97.4	A
CZ10_09	**99.99 / 99.7**	99.95 / 98.3	99.92 / 97.3	99.94 / 98.2	A
NA1	99.85 / 95.5	99.91 / 97.2	99.91 / 97.3	99.94 / 98.1	Unidentified
NA2	99.88 / 96.1	99.89 / 96.4	99.93 / 97.8	99.92 / 97.4	Unidentified
NA3	99.80 / 93.7	99.87 / 96.0	**99.99 / 99.8**	**99.99 / 99.7**	C,D
NA4	99.74 / 92.1	99.88 / 96.3	99.78 / 93.2	**99.99 / 99.6**	D
NA5 branch 1	99.80 / 93.9	99.88 / 96.1	99.90 / 97.0	**99.99 / 99.6**	D
NA5 branch 2	99.83 / 94.5	99.89 / 96.5	99.93 / 97.7	**99.99 / 99.6**	D
NA6	99.81 / 94.1	99.87 / 96.0	99.86 / 95.6	**99.99 / 99.6**	D
NA7	99.79 / 93.6	99.86 / 95.7	**99.99 / 99.5**	99.91 / 97.3	C
Durum	99.78 / 93.1	99.66 / 89.9	99.77 / 92.8	99.67 / 90.0	Unidentified
Middle East (ISR173B)	99.79 / 93.6	99.91 / 97.0	99.78 / 93.0	99.86 / 95.7	Unidentified
Other European isolates	99.87 / 96.1	99.92 / 97.6	99.85 / 95.5	99.89 / 96.4	Unidentified

We report both the fraction of bases in the *k*-mers that are shared between the genome and the sequencing reads (termed ‘*k*-mer identity’) and the *k*-mers that are shared between the genome and the sequencing read set (termed ‘shared *k*-mers). Bold entries indicate a haplotype genome considered to be fully contained in the sequencing reads of an isolate (*k-*mer identity ≥99.99%, shared *k*-mers ≥99.5%).

### Hybrid lineages of *Pt* have spread worldwide

Because the whole-genome sequence data used above are biased towards North American and Australian isolates, we combined this with a restriction site-associated genotyping by sequencing (GBS) SNP analysis of 559 isolates representing 11 global regions (North America, South America, Middle East, Central Asia, Europe, East Africa, Russia, China, Pakistan, New Zealand and South Africa)^35^. A phylogenetic tree constructed from this data (Extended Data Fig. 9) showed an overall similar topology to the whole-genome tree (Fig. 3) for the isolates and clades common to both data sets, confirming that this analysis with a reduced SNP set is robust.

**Extended Data Fig. 2:**
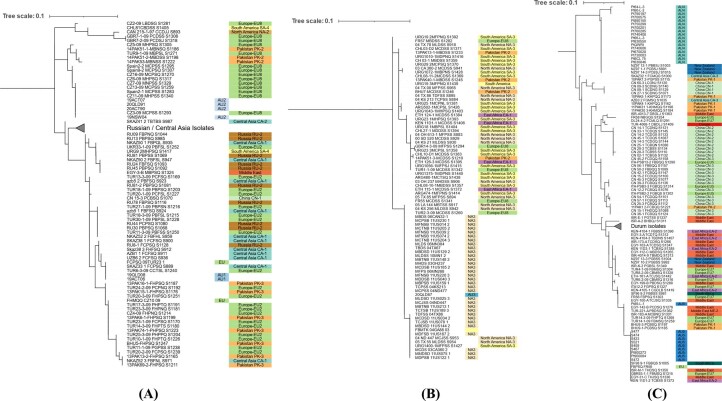
Pruned phylogenetic trees of SNP genotypes shared between GBS and whole-genome data. The left color strip indicates which isolates have whole-sequencing data and their lineage assignment. The right color strip indicates which isolates have GBS data and their lineage assignment^35^. **(a)** The part of the tree that contains the Australian AU1 isolates also contains the EU2 lineage as well as clades from Central Asia and Pakistan, suggesting this lineage is common to this broad geographical region. The Australian AU2 isolates cluster with one group of the European clade EU8. **(b)** The Australian AU3 isolate is again placed with the North American clade NA3. **(c)** The Australian AU5 isolates group with the EU7 lineage, whereas the AU4 isolates group only with three isolates from New Zealand. Full tree is shown in Extended Data Fig. 9.

This expanded phylogenetic tree places the AU1 isolates (AB haplotype) into a clonal clade containing all 19 isolates of the EU2 lineage, including 09TUR23-1 (Extended Data Fig. 2a). Although these EU2 isolates were collected in 2009, previous studies identified isolates of this pathotype group in Europe in the 1990s (refs. ^36–38^), suggesting that it was present before its first detection in Australia in 2005. In addition, isolates of the Central Asian clade CA1 and Pakistan clade PK3 collected in 2002 and 2003 fall within this lineage group, suggesting that the AB genotype lineage is common to Europe, Asia and Australasia. The AU3/NA3 clonal group included isolates from the European EU8 (5 isolates) and South American SA3 clades (22 isolates) (Extended Data Fig. 2b), indicating that the CD genotype lineage is common to the Americas, Europe and Australasia. However, the previously defined EU8 clade is split into two groups in this tree, with the second group (11 isolates) forming a clonal group with the AU2 (BC) isolates and some isolates from Pakistan (PK-2 clade). The shared C genome between these two EU8 subgroups may explain why they were not separated previously on the basis of simple sequence repeat (SSR) analysis^11^. Importantly, this suggests that there may have been hybrid BC haplotype isolates in Europe in 2009 before they were detected in Australia, indicating either independent hybridization events in both continents or migration of a hybrid strain from Europe to Australia. This tree also supports a clonal relationship between the AU5 group and all eight isolates of the EU7 clade, confirming the relationship seen with the single EU7 isolate by FR56 (Fig. 3), consistent with introduction of this A haplotype-containing lineage from Europe to Australia (Extended Data Fig. 2c).

### Genetic diversity of the mating type loci in *Pt*

**Extended Data Fig. 3:**
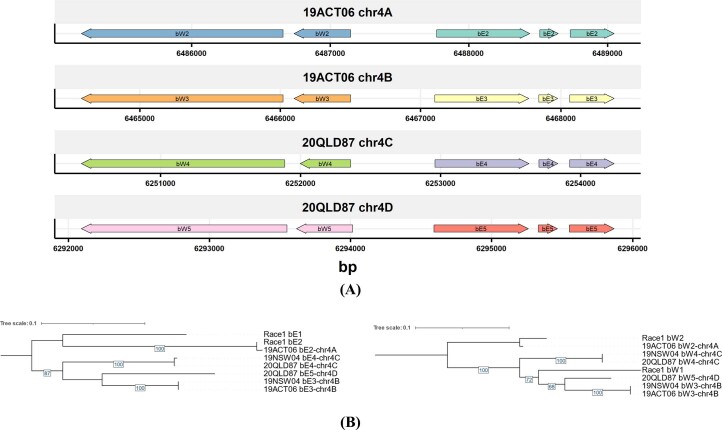
Chromosome location and phylogenetic trees of the mating type proteins bE and bW. **(a)** Five variants of the *b* genes are present in the listed *Pt* isolates on chromosome 4 and in the race 1 reference genome^40^. **(b)** The b2 proteins in race 1 are shared with the 19ACT06 haplotype A. Bootstrap values are shown.

**Extended Data Fig. 4:**
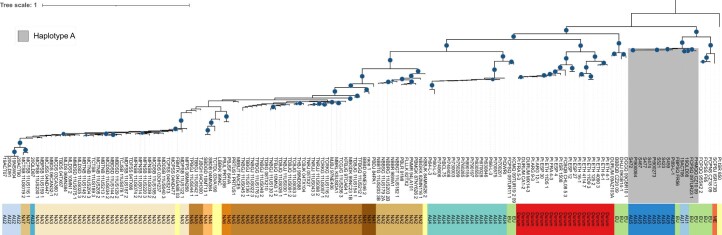
Haplotype-specific phylogeny of global isolates. Phylogenetic tree of global isolates against 19ACT06 haplotype A.

**Extended Data Fig. 5:**
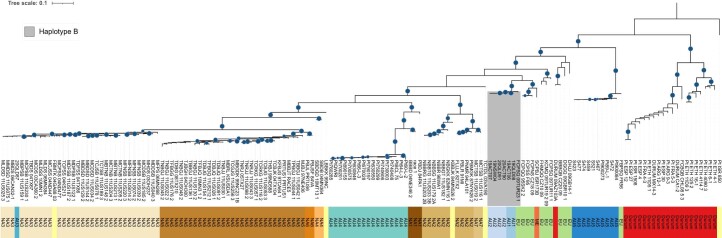
Haplotype-specific phylogeny of global isolates. Phylogenetic tree of global isolates against 19NSW04 haplotype B.

**Extended Data Fig. 6:**
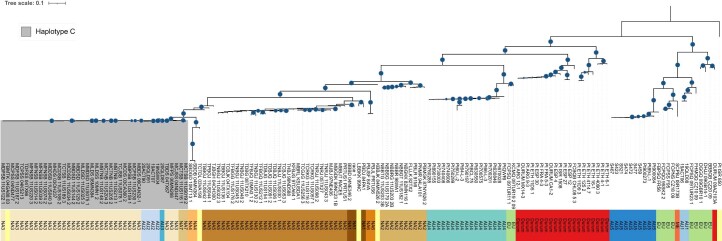
Haplotype-specific phylogeny of global isolates. Phylogenetic tree of global isolates against 20QLD87 haplotype C.

**Extended Data Fig. 7:**
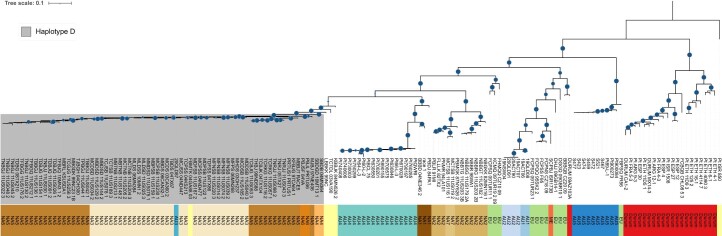
Haplotype-specific phylogeny of global isolates. Phylogenetic tree of global isolates against 20QLD87 haplotype D.

**Extended Data Fig. 8:**
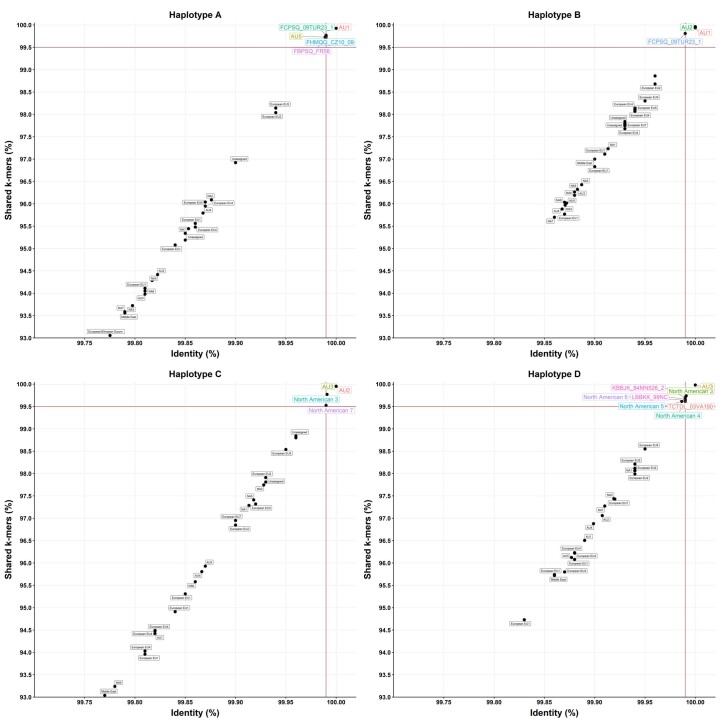
*k*-mer genome containment scores of Illumina sequencing reads against the four haplotypes. Identity is the percentage of bases that are shared between the genome and the sequencing reads. Shared *k-*mers is the percentage of *k*-mers shared between the genome and the sequencing reads. Two red lines indicate above which thresholds we consider a haplotype genome to be fully contained in the sequencing reads of an isolate (identity >= 99.99%, shared *k*-mers >= 99.5%). The averages are shown for clades.

Mating compatibility in many basidiomycetes is controlled by two loci. The *a* locus encodes a pheromone/receptor pair and the *b* locus encodes two homeodomain transcription factors, bEast and bWest (bE and bW)^39^. However, the role of these loci in either sexual or asexual compatibility in rust fungi has not been directly determined. Two alleles (+ and −) of the *a* locus receptor (STE3.2 and STE3.3 genes, respectively) are present in each of the *Pt* genome assemblies on chromosome 9, with the B and D haplotypes encoding identical + alleles and the A and C haplotypes the − allele. Whole-genome SNP data showed that all 154 *Pt* isolates contain both alleles with no more than one or two SNPs in either gene (Supplementary Data 1). The universal heterozygosity of these two alleles is consistent with successful dikaryon formation after somatic hybridization, requiring the presence of different *a* locus alleles in the two nuclear haplotypes. In contrast, multiple divergent alleles of the *b* locus on chromosome 4 were detected, with the A haplotype containing the same *b2* allele defined from de novo RNAseq assemblies in race 1 (ref. ^40^) and the B, C and D haplotypes containing additional allelic variants designated as *b3*, *b4* and *b5*, respectively (Extended Data Fig. 3 and Supplementary Fig. 8). SNP calling against the 19ACT06 (genotype *b2*/*b3*) and 20QLD87 (*b4*/*b5*) diploid reference genomes, as well as *k*-mer containment analysis, confirmed that isolates sharing the A, B, C or D haplotypes contain the same *b* locus alleles (*b2* to *b5*) as these reference haplotypes (Supplementary Table 7), in some cases along with an additional undefined divergent allele.

## Discussion

**Extended Data Fig. 9:**
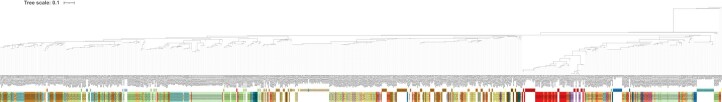
Full phylogenetic trees of SNP genotypes shared between GBS and whole-genome data. The top color strip indicates which isolates have whole-sequencing data and their lineage assignment. The bottom color strip indicates which isolates have GBS data and their lineage assignment^35^.

*Pt* is a widely distributed fungus that shows asexual reproduction in most parts of the world^8^, with a number of clonal groups common to Europe, Asia, the Americas and Africa^34,35^. Although somatic genetic exchange between rust strains was well established in laboratory infections, its contribution to population diversity in the field has been largely unknown and debates over whether such exchanges involved transfer of whole nuclei or parasexual recombination remain unresolved. Here we found by nuclear haplotype comparisons that extensive nuclear exchange events without recombination have occurred in natural populations of the wheat leaf rust fungus *Pt* and have given rise to many of the long-term clonal lineages of this pathogen common around the world (Fig. 5). Whole-genome comparison of haplotype-resolved assemblies showed that the most recently emerged Australian lineage, AU2 (BC nuclear genotype), is derived by nuclear exchange between members of the AU1 (AB) and AU3 (CD) lineages, representing the European and North American lineages EU2 and NA3, respectively. Haplotype-specific phylogenetic and *k*-mer containment analysis further revealed numerous nuclear exchange events between major clonal lineages. For instance, the NA3 group (CD), which was first detected in 1996 as a newly emerged pathotype with a novel virulence combination^41^, most probably arose from a nuclear exchange event involving an isolate of the NA5 group donating the D genome. The NA4, NA5 and NA6 lineages all share the D haplotype, and include isolates collected in the 1950s and 1960s (refs. ^9,34^), with similar pathotypes first described in 1920/1921 (ref. ^42^), suggesting that these lineages were already prevalent a hundred years ago. The NA5 lineage also occurred in South America dating back to at least 1981 (refs. ^10,35^), while NA3 isolates were first detected there in 1999. Thus, our data are consistent with a proposal^10^ that the NA3 group migrated to the Northern US and South America from Mexico, where similar pathotypes had been detected earlier in the 1990s, making this a likely location for the hybridization event giving rise to NA3. Two other North American isolates (NA7) contain the C haplotype and could represent the other parental lineage of NA3, as suggested by their basal position to NA3 in the C haplotype phylogeny (Fig. 4c). However, it is also possible that they are derived from NA3 given their isolation in 2011. NA3 is now the most commonly isolated pathotype group in US surveys^12^, and some isolates from Europe (collected in 2004 and 2009) and Pakistan (2010–2014) clustered with NA3 by both SSR and GBS SNP genotypes (Extended Data Figs. 2 and 9), indicating that this hybrid lineage has spread worldwide.

In addition to the above, two European lineages (EU5 and EU7, the latter including the Australian AU5 lineage) contain the A haplotype, expanding the set of global lineages related by nuclear exchange events to at least 13 of the 17 major lineages examined here. Thus, these events seem to be very common in global populations of *Pt*, which are dominated by isolates with different combinations of a relatively small number of haploid genotypes. Given this high frequency of nuclear exchange, there is the potential for repeated shuffling of haplotypes within populations re-creating the same haplotype combinations in different locations or times. For example, Fig. 4 suggests that one branch of the NA5 group may contain a D nucleus derived from the NA3 (CD) group by a subsequent hybridization event, effectively exchanging related but slightly diverged D genomes between these lineages. Indeed, the NA3 lineage phylogenies derived from the C and D haplotypes (Fig. 4c,d) show several incongruities, which could occur if members of this lineage have undergone repeated exchanges of the C and D nuclei. Furthermore, Extended Data Fig. 2a suggests that some European isolates from 2009 are closely related to the AU2 (BC) group, suggesting that this haplotype combination may have been generated independently in Europe and Australia, although it is also possible that the hybrid lineage originated in Europe and coincidently migrated to Australia after the AB and CD parental linages.

Early studies on laboratory-induced somatic exchange resulted in competing hypotheses involving exchange of intact nuclei of opposite mating types (*M. lini*, *P. coronata* f. sp. *avenae* and *P. recondita*)^17,18^ or parasexual recombination (*P. recondita*)^43^. Hybrids obtained in flax rust *M. lini* contained only parental nuclear combinations of alleles of several *Avr* loci, consistent with the former hypothesis, but the lack of molecular markers meant that this could not be resolved in other rust fungi. The haplotype-specific genome data here show clearly that no recombination occurred in the generation of the BC genotype (AU2 lineage) in either of the parental isolates before donation of their nuclei, or in the hybrid line subsequent to the exchange event. Likewise, the presence of the C and D haplotypes in separate nuclei of 20QDL87, along with the entire D haplotype in NA4 and NA6, indicates no recombination either in the parental or hybrid isolates. Similar considerations apply to the shared A haplotype in AU1, EU5 and EU7/AU5, as well as the hybrid lineages of *Pgt* including Ug99 (ref. ^22^). All of the *Pt* isolates related by hybridization contain two opposite alleles (+/−) at the *a* mating type locus, consistent with this being a requirement for a viable hybrid. Thus, it appears that somatic hybridization in *Pt* and *Pgt* typically involves whole nuclear exchange without recombination. The high impact of nuclear exchange in these species may be a consequence of the absence of sexual hosts in most wheat growing areas, resulting in populations consisting of long-lived clonal lineages.

Generating haplotype-phased genome references for additional global rust isolates will help to confirm the proposed origins of nuclear haplotypes and identify other prevalent haplotypes. The latest version of hifiasm^30^ incorporating Hi-C data into PacBio HiFi assembly greatly facilitates rapid generation of accurate nuclear haplotypes, with only three phase-switch artefacts detected by NuclearPhaser^24^ across the three raw *Pt* genome assemblies. This compares to 31 and 33 phase-switch contigs in the PacBio Canu assemblies of *Pgt*21-0 and *Pca*203, respectively^22,27^, and 14 and 17 phase-switch contigs in the PacBio-HiFi assemblies of 19ACT06 using hifiasm (without Hi-C data) and HiCanu, respectively^24^. FALCON-Phase^44^ can also incorporate Hi-C data, but a chromosome-level haplotype-separated assembly generated for *Pt*64 (ref. ^45^) with this assembler was not assessed for potential phase switches and chromosomes were assigned to pseudo-haplotypes without using Hi-C contact information, which may therefore contain chromosomes from each nucleus. Another *Pt* isolate chromosome-scale reference was assembled using Hi-C reads from *Pt*76 and *Pt*64 and is thus not phased^46^.

Although duplicated pycnial fertilization events during sexual reproduction could give rise to progeny sharing a single common nucleus^47^, this is an untenable explanation for the multiple haplotypes shared between global lineages in *Pt* and *Pgt*^22^ since it requires that all such lineages were generated by simultaneous cross-fertilization events. This is not consistent with the recent emergence of the CD and BC lineages of *Pt* in the 1990s and 2010s, compared with the NA4, NA5 and NA6 lineages dating back over 100 yr. The phylogenetic data also support different divergence times of the common haplotypes in these lineages, rather than divergence from a single common ancestor. Similar observations apply to shared haplotypes in *Pgt* lineages^22^.

## Methods

### Sampling and pathotyping of the *Pt* isolates

Rust-infected samples from wheat cultivar Morocco were collected in 2019 and 2020 from the CSIRO field site in Canberra, Australian Capital Territory (19ACT07 and 20ACT90) and from the wheat cultivar Grenade in a field at the Department of Primary Industries, Wagga Wagga, New South Wales (19NSW04). Three samples were collected in 2019/20 from an unknown wheat cultivar in Warwick, Queensland (20QLD87) or Gatton, Queensland (19QLD08 and 20QLD91). The 19ACT06 isolate was sampled as previously described^24^. *Pt* cultures were purified through single pustule isolation and pathotyped using the standard Australian wheat differential sets carrying unique resistance genes and nomenclature for leaf rust^48^ (Supplementary Table 8).

### PacBio HiFi DNA and Hi-C sequencing

High molecular DNA from urediniospores was extracted as previously described^22,49^. DNA quality was assessed with a Nanodrop spectrophotometer (Thermo Scientific) and the concentration quantified using a broad-range assay in a Qubit 3.0 fluorometer (Invitrogen). DNA library preparation (10–15 kb fragments Pippin Prep) and sequencing in PacBio Sequel II Platform (One SMRT Cell 8M) were performed by the Australian Genome Research Facility (AGRF) (St Lucia, Queensland, Australia) following manufacturer guidelines. For DNA crosslinking and subsequent Hi-C sequencing, 100 mg of urediniospores was suspended in 4 ml 1% formaldehyde, incubated at r.t. for 20 min with periodic vortexing. Glycine was added to 1 g per 100 ml and the suspension was centrifuged at 1,000 *g* for 1 min and the supernatant was removed. Spores were then washed with H_2_O, centrifuged at 1,000 *g* for 1 min and the supernatant removed. The spores were then transferred to a liquid nitrogen-cooled mortar and ground before being stored at −80 °C or on dry ice. After treatment, spores were shipped to Phase Genomics (Seattle, Washington, USA) for Hi-C library preparation and sequencing.

### Illumina short-read whole-genome sequencing of *Pt* isolates

Genomic DNA was extracted from 30 mg of urediniospores per isolate using the Omniprep DNA isolation kit (G-Biosciences). DNA concentration was determined using a Qubit 3.0 fluorometer (LifeTechnologies) before submission for whole-genome sequencing. A transposase-based library was prepared for each sample with DNA Prep (M) tagmentation kit (Illumina) at the AGRF following manufacturer guidelines. DNA sequencing was completed at AGRF using a NovaSeq S4, 300 cycles platform (Illumina) to produce 150 bp paired-end reads.

### Genome assembly and scaffolding

The HiFi reads of the isolates 19NSW04 and 20QLD87 were assembled using hifiasm 0.16.1 in Hi-C integration mode and with default parameters (19NSW04: 15.2 Gb HiFi reads and 34.8 Gb Hi-C reads; 20QLD87: 12.3 Gb HiFi reads and 43.9 Gb Hi-C reads)^30^. Contaminants were identified using sequence similarity searches (BLAST 2.11.0 -db nt -evalue 1e-5 -perc_identity 75) (ref. ^50^). HiFi reads were aligned to the assembly with minimap2 2.22 (-ax map-hifi –secondary=no)^51^ and contig coverage was called using bbmap’s pileup.sh tool on the minimap2 alignment file (http://sourceforge.net/projects/bbmap/). All contaminant contigs, contigs with less than 5x coverage and the mitochondrial contigs were removed from the assembly. BUSCO completeness was assessed with v.3.0.2 (-l basidiomycota_odb9 -sp coprinus) and Augustus parameters pre-trained on the *Pt*76 (19ACT06) assembly^52^. The HiFi reads of *Pt*76 isolate were re-assembled using hifiasm 0.16.1 in Hi-C integration mode and with default parameters to assess improvement in phasing compared to the previously published HiCanu assembly^24^.

Phasing of the assembled haplotypes was confirmed using the NuclearPhaser pipeline v.1.1 (MAPQ = 30; https://github.com/JanaSperschneider/NuclearPhaser)^24^. Hi-C data provide a strong nuclear origin signal reflecting the physically separate nuclei in the dikaryon, with ~90% of *trans* and >99% of *cis* and *trans* Hi-C links occurring within a nucleus in the *Pt* assemblies, similar to those of *Pgt* and *Pca*^22,27^ as well as other fungal dikaryons^31^. The low level (<10%) of Hi-C *trans* read pairs mapping across haplotypes could result from disruption of some nuclei during chromatin crosslinking, ligation of non-crosslinked DNA fragments or mapping of reads to haplotype-collapsed or highly similar regions (Supplementary Fig. 2b).

The HiFi reads of the 20QLD87 isolate were also assembled with HiCanu 2.2.0 to confirm phase-switch boundaries (genomeSize=120 m -pacbio-hifi)^29^, and contigs were aligned to the hifiasm assembly with minimap2 (ref. ^51^). Per-base consensus quality scores for the assemblies were obtained using Merqury (1.3) (ref. ^53^).

We curated nuclear-phased chromosomes for each assembly by scaffolding the two haplotypes separately and then further joined scaffolds into chromosomes through visual inspection of Hi-C contact maps. For scaffolding of the individual haplotypes, the Hi-C reads were mapped to each haplotype using BWA-MEM (0.7.17) (ref. ^54^) and alignments were then processed with the Arima Genomics pipeline (https://github.com/ArimaGenomics/mapping_pipeline/blob/master/01_mapping_arima.sh). Scaffolding was performed using SALSA (2.2) (ref. ^55^). Hi-C contact maps were produced using Hi-C-Pro 3.1.0 (MAPQ = 10) (ref. ^56^) and Hicexplorer (3.7.2) (ref. ^57^).

### Gene prediction and repeat annotation

De novo repeats were predicted with RepeatModeler 2.0.2a and the option -LTRStruct^58^. RepeatMasker 4.1.2p1 (-s -engine ncbi) (http://www.repeatmasker.org) was run with the RepeatModeler library to obtain statistics about repetitive element content. For gene prediction, RepeatMasker was run with the RepeatModeler library and the options -s (slow search) -nolow (does not mask low_complexity DNA or simple repeats) -engine ncbi. RNAseq reads from *Pt*76 (ref. ^24^) were aligned to the genome using HISAT2 2.1.0 (–max-intronlen 3000 –dta)^59^, and genome-guided Trinity 2.8.4 (–jaccard_clip –genome_guided_bam –genome_guided_max_intron 3000) was used to assemble transcripts^60^. We then aligned each RNAseq sample to the individual haplotype chromosomes as well as the unplaced contigs using HISAT2 (v.2.1.0 –max-intronlen 3000 –dta)^59^. We used StringTie 2.1.6 (-s1 -m50 -M1) to assemble transcripts for each sample^61^. The transcripts of the ungerminated and germinated spore samples were merged into a spore transcript set for each haplotype chromosome as well as the unplaced contigs using StringTie (–merge). The transcripts of the infection timepoint samples were merged into an infection transcript set for each haplotype chromosome as well as the unplaced contigs using StringTie (–merge).

Funannotate (1.8.5) (ref. ^62^) was run to train PASA (funannotate update) with the preassembled Trinity transcripts as input^63^. CodingQuarry (2.0) (ref. ^64^) was run in pathogen mode, once on the infection transcripts and once on the spore transcripts. For the infection transcripts, we merged the predicted genes, the predicted pathogen genes and the predicted dubious gene set into the final CodingQuarry infection gene predictions. For the spore transcripts, we merged the predicted genes, the predicted pathogen genes and the predicted dubious gene set into the final CodingQuarry spore gene predictions. We then ran funannotate predict (–ploidy 2 –optimize_augustus –busco_seed_species ustilago –weights pasa:10 codingquarry:0) and supplied Trinity transcripts and *Pucciniomycotina* EST clusters downloaded from the JGI MycoCosm website (http://genome.jgi.doe.gov/pucciniomycotina/pucciniomycotina.info.html). We also supplied our CodingQuarry predictions to funannotate with the option -other_gff and set the weight of the CodingQuarry infection gene predictions to 20 and the weight of the CodingQuarry spore gene predictions to 2. After the funannotate gene predictions, we ran funannotate update followed by an open reading frame (ORF) prediction to capture un-annotated genes that encode secreted proteins. First, we ran TransDecoder 5.5.0 (https://github.com/TransDecoder/TransDecoder) on the StringTie infection transcripts (TransDecoder.LongOrfs -m50 and TransDecoder.Predict –single_best_only). We selected ORFs that have a start and stop codon (labelled as ‘complete’) and predicted those that have a signal peptide (SignalP 4.1 -u 0.34 -U 0.34) and no transmembrane domains outside the N-terminal signal peptide region (TMHMM 2.0) (refs. ^65,66^). We added genes encoding secreted proteins to the annotation using agat_sp_fix_overlaping_genes.pl^67^, which creates isoforms for genes with overlapping coding sequence. In line with funannotate, we did not include genes encoding secreted proteins that are >90% contained in a repetitive region in the final annotation. Functional annotation of proteins was predicted using InterProScan (5.56–89.0) (ref. ^68^).

### Genome comparisons and *k*-mer containment screening

The haplotype chromosomes were compared to each other with mummer 4.0.0rc1, using nucmer and dnadiff^69^. The dnadiff VCF files were used in SNPeff 5.1 to assess the impact of variants on coding regions^70^. Genomic dot plots were produced using D-GENIES^71^. Mash (2.3) (ref. ^72^) was used for *k*-mer containment screening (mash screen with sketch settings -s 500000 -k 32). Mash returns both the fraction of bases in the *k*-mers that are shared between the genome and the sequencing reads (termed ‘*k*-mer identity’) and the *k*-mers that are shared between the genome and the sequencing read set (termed ‘shared *k*-mers). We also calculated the averages of *k*-mer containment across clades. Genome plots were drawn using karyoploteR and gggenes (https://wilkox.org/gggenes/).

### Phylogenetic trees and mating type loci

Illumina reads were downloaded from NCBI and cleaned with trimmomatic (v.0.38) (ref. ^73^) and then aligned against the diploid chromosome assemblies using BWA-MEM (0.7.17) (ref. ^54^). The alignment files of our seven isolates were filtered for minimum quality 30 as the coverage was substantially higher than for the alignments of the other global isolates. SNPs were called using FreeBayes 1.3.5 (–use-best-n-alleles 6 –ploidy 2) in parallel mode^74^ against the diploid chromosomes and the individual haplotype chromosomes. SNPs were filtered using vcffilter of VCFlib 1.0.1 (https://github.com/vcflib/vcflib) with the parameter -f ‘QUAL > 20 & QUAL / AO > 10 & SAF > 0 & SAR > 0 & RPR > 1 & RPL > 1 & AC > 0’. Bi-allelic SNPs were selected using vcftools (–min-alleles 2 –max-alleles 2 –max-missing 0.9 –maf 0.05) (ref. ^75^) and converted to multiple sequence alignment in PHYLIP format using the vcf2phylip script^76^. Phylogenetic trees were constructed using RAxML (8.2.12)^77^. We generated 500 bootstrap trees (-f a -# 500 -m GTRCAT) and a maximum-likelihood tree (-D), and incorporated these models into a final tree (-f b -z -t -m GTRCAT). Phylogenetic trees were visualized in iTOL (v.6) (ref. ^78^) with the isolate ISR850 as the outgroup. We used the publicly available GBS SNP vcf file (https://conservancy.umn.edu/handle/11299/208672) and the genomic Illumina data to build a phylogenetic tree as follows. Since the GBS SNP data were derived from mapping to the *Pt* ASM15152v1 draft assembly^35^, we mapped the whole-genome sequence data of the 154 isolates onto this reference and extracted SNP genotypes on a set of 631 polymorphic sites that were represented in both data sets. First, we mapped the clean Illumina reads to the *Pt* ASM15152v1 draft assembly downloaded from https://fungi.ensembl.org/Puccinia_triticina/Info/Index^35^. SNPs were called as described above. We intersected the GBS SNP vcf file with the Illumina SNP file using bcftools isec^79^ and kept SNPs shared by both files. Phylogenetic trees were constructed as described above.

The b1–b5 proteins were aligned using mafft (7.4.90) (ref. ^80^) and a phylogenetic tree was built using iqtree2 2.2.0.8 (-B 1000 -alrt 1000) (ref. ^81^), visualized and midpoint-rooted in iTOL (v.6) (ref. ^78^). SNP statistics were collected using bcftools stats and coverage statistics using samtools coverage^79^.

### Reporting summary

Further information on research design is available in the Nature Portfolio Reporting Summary linked to this article.

### Supplementary information

Supplementary Information Supplementary tables and figures.

Reporting Summary

Supplementary Data 1–3 Supplementary Data 1. Effects of SNPs on coding genes. 2. Isolate information and mating type allele analysis. 3. *k*-mer containment analysis data.

## Data Availability

All sequence data and assemblies generated in this study are available at NCBI BioProject PRJNA902835. Sequencing reads, assemblies and gene annotation files are also available at the CSIRO Data Access Portal (https://data.csiro.au/collection/csiro:57097).
