# Supplementary material for: Nuclear exchange generates population diversity in the wheat leaf rust pathogen Puccinia triticina
Source: Nat Microbiol. 2023 Oct 26;8(11):2130–41. doi: 10.1038/s41564-023-01494-9 (PMC10627818; doi:10.1038/s41564-023-01494-9)
Supplement: Supplementary file 1 — Supplementary tables and figures. [file 41564_2023_1494_MOESM1_ESM.pdf]

nature microbiology

Article

https://doi.org/10.1038/s41564-023-01494-9

Nuclear exchange generates population diversity in the wheat leaf rust pathogen *Puccinia triticina*

In the format provided by the authors and unedited

Supplementary Table S1: The *Pt* isolates collected in this study with sample locations in Australia and pathotypes. The nuclear genotypes derived from *k*-mer and lineage assignments according to phylogenetic tree analysis are shown. The nuclear genotype of 20QLD87 later derived as CD is also shown. (ACT: Australian Capital Territory; QLD: Queensland; NSW: New South Wales).

Isolate name	Sample collection	Collection year	Pathotype	Nuclear genotype	Lineage
19ACT06 (*Pt76*) [24]	Canberra, ACT	2019	76-3,5,7,9,10,12,13	AB	AU1
19QLD08	Gatton, QLD	2019	76-1,3,5,7,9,10,12,13	AB	
19NSW04	Wagga Wagga, NSW	2019		BC	AU2
19ACT07	Canberra, ACT	2019	104-1,3,4,5,6,7,9,10,12	BC	
20QLD91	Gatton, QLD	2020		BC	
20ACT90	Canberra, ACT	2020	104-1,3,4,5,7,9,10,12	BC	
20QLD87	Warwick, QLD	2020	104-1,3,4,6,7,8,9,10,12	CD	AU3

Supplementary Table S2: Statistics for the hifiasm-haplotype 1 and hifiasm-haplotype 2 assemblies before scaffolding.

Statistic	19NSW04 hifiasm-haplotype 1	19NSW04 hifiasm-haplotype 2	20QLD87 hifiasm-haplotype 1	20QLD87 hifiasm-haplotype 2
Assembly size	129.487 Mb	123.558 Mb	123.181 Mb	125.281 Mb
# of contigs	119	61	81	64
N/L50	8/6.813 Mb	9/6.623 Mb	9/5.992 Mb	9/5.943 Mb
Maximum scaffold length	9.616 Mb	8.623 Mb	8.313 Mb	8.988 Mb
GC content	46.54%	46.57%	46.59%	46.62%
Complete BUSCOs (%)	96.3%	95.2%	95.7%	96.3%
Duplicated BUSCOs (%)	5.4%	3.9%	3.6%	4.2%
Fragmented BUSCOs (%)	2.7%	2.6%	3%	2.7%
Haplotype identity	C	B	D	C

Supplementary Table S3: Assembly statistics for the two haplotypes of the scaffolded hifiasm assemblies with Hi-C integration.

Statistic	19NSW04 haplotype C chromosomes	19NSW04 haplotype B chromosomes	19NSW04 unplaced contigs	20QLD87 haplotype D chromosomes	20QLD87 haplotype C chromosomes	20QLD87 unplaced contigs
Assembly size	123.8 Mb	122.1 Mb	7.2 Mb	121.8 Mb	123.3 Mb	3.0 Mb
Number of scaffolds/contigs	18	18	123	18	18	79
N/L50	-	-	27/76.2 Kb	-	-	21/38.7 Kb
Maximum scaffold length	9.6 Mb	9.2 Mb	326.9 Kb	9.3 Mb	9.4 Mb	164.3 Kb
GC content	46.6%	46.7%	43.6%	46.6%	46.6%	44.1%
Complete BUSCOs (%)	96.1%	96.2%	0.8%	96%	96.1%	0.2%
Duplicated BUSCOs (%)	3.7%	3.5%	0.1%	3.4%	3.6%	0.1%
Fragmented BUSCOs (%)	2.8%	2.7%	0.1%	2.8%	2.8%	0%
Number of genes	17,939	18,138	245	18,952	18,804	90
% repetitive sequence	63.9%	63.5%	92.7%	62.6%	63.1%	90%
% retroelements	31.4%	30.9%	15.8%	31.1%	31.9%	19.7%
% DNA transposons	6.3%	6.4%	1.4%	6.4%	6.5%	3.3%
% unclassified repeats	23.6%	24%	19.5%	23.6%	23.2%	24%
% ribosomal RNAs	0.9%	0.7%	51.6%	0.6%	0.6%	40.3%
Assembly consensus quality value (QV score)		55.6	-		59.7	-

Supplementary Table S4: Statistics for genomic alignments between the *Pt76* and the 19NSW04/20QLD87 haplotype chromosomes.

Within-isolate alignments	**19NSW04 haplotype B versus haplotype C**	**20QLD87 haplotype C versus haplotype D**	***Pt76* haplotype A versus haplotype B**
Aligned bases	97.9%	97.4%	97.1%
Average identity of 1-to-1 alignments	99.5%	99.5%	99.5%
Average identity of M-to-M alignments	99.1%	99.1%	99%
Translocations	849	848	1,053
Inversions	155	132	164
Insertions	8,913	8,617	10,898
Total SNPs	328,470	301,814	334,189
Total Indels	182,991	167,011	186,643

Between-isolate alignments	***Pt76* haplotype B versus 19NSW04 haplotype 1**	***Pt76* haplotype B versus 19NSW04 haplotype 2**	**20QLD87 haplotype 2 versus 19NSW04 haplotype 2**	**20QLD87 haplotype 2 versus 19NSW04 haplotype 1**	**20QLD87 haplotype 1 versus *Pt76* haplotype A**
Aligned bases	99.03%	**99.97%**	98.88%	**99.98%**	98.76%
Average identity of 1-to-1 alignments	99.49%	**99.98%**	99.49%	**99.99%**	99.49%
Average identity of M-to-M alignments	99.06%	**99.95%**	99.07%	**99.97%**	99.07%
Translocations	900	**1**	862	**4**	986
Inversions	157	**6**	145	**2**	146
Insertions	10,601	**347**	11,233	**260**	11,544
Total SNPs	325,608	**2,966**	328,689	**2,182**	327,782
Total Indels	181,972	**9,457**	182,772	**6,486**	180,947
**Assignment**		19NSW04 haplotype 2 = B		19NSW04 haplotype 1 = C 20QLD87 haplotype 2 = C	

Supplementary Table S5: SNPs derived from genomic alignments and their properties.

**Comparison**	**Number of SNPs**	**SNPs in repetitive regions**	**SNPs in coding regions**	**Non-synonymous SNPs**	**Nonsense SNPs**	**Number of proteins with variant**	**Number of secreted proteins with variant**
19ACT06 B - 19NSW04 B	2,966	2,367 (80.1%)	283 (9.5%)	163 (57.6%)	7 (2.5%)	60	5 (8.3%)
19NSW04 C – 20QLD87 C	2,182	1,773 (81.3%)	178 (8.2%)	100 (56.2%)	3 (1.7%)	122	21 (17.2%)
19ACT06 A - 19ACT06 B	334,203	262,845 (78.7%)	35,110 (10.5%)	21,232 (60.5%)	526 (1.5%)	7,630	1,215 (15.9%)
19NSW04 B - 19NSW04 C	328,470	256,429 (78.1%)	34,941 (10.6%)	21,340 (61.1%)	474 (1.4%)	7,858	1,223 (15.6%)
20QLD87 C - 20QLD87 D	301,814	258,447 (85.6%)	32,754 (10.9%)	20,114 (61.4%)	529 (1.6%)	7,384	1,108 (15%)

Supplementary Table S6: SNPs derived from genomic alignments and their location on the chromosomes.

B haplotype variants				C haplotype variants			
**Chromosome (19NSW04)**	**Length**	**Variants**	**Variants rate**	**Chromosome (19NSW04)**	**Length**	**Variants**	**Variants rate**
1_B	9,204,362	158	58,255	1_C	9,316,129	94	99,107
2_B	7,951,971	112	70,999	2_C	7,154,143	26	275,159
3_B	8,920,942	82	108,791	3_C	9,016,186	39	231,184
4_B	8,537,679	259	32,964	4_C	8,922,052	100	89,220
5_B	8,007,851	259	30,918	5_C	7,944,558	162	49,040
6_B	8,182,878	98	83,498	6_C	9,615,590	66	145,690
7_B	7,671,061	360	21,308	7_C	7,664,942	60	127,749
8_B	7,350,761	108	68,062	8_C	7,484,054	37	202,271
9_B	6,788,249	93	72,991	9_C	7,031,919	26	270,458
10_B	7,565,484	368	20,558	10_C	7,216,867	35	206,196
11_B	6,990,834	106	65,951	11_C	6,813,017	281	24,245
12_B	5,939,218	118	50,332	12_C	6,034,179	227	26,582
13_B	5,575,677	231	24,137	13_C	5,707,219	23	248,139
14_B	5,329,819	49	108,771	14_C	5,388,068	47	114,639
15_B	4,442,786	63	70,520	15_C	4,475,553	23	194,589
16_B	4,691,570	253	18,543	16_C	4,941,809	39	126,713
17_B	4,550,902	197	23,101	17_C	4,705,371	64	73,521
18_B	4,374,653	52	84,127	18_C	4,313,118	833	5,177
**Total**	**122,076,697**	**2,966**	**41,158**	**Total**	**123,744,774**	**2,182**	**56,711**

AB haplotype variants				BC haplotype variants			
**Chromosome (19ACT06)**	**Length**	**Variants**	**Variants rate**	**Chromosome (19NSW04)**	**Length**	**Variants**	**Variants rate**
1_A	9,506,148	22,218	427	1_B	9,204,362	21,390	430
2_A	8,009,403	25,043	319	2_B	7,951,971	20,565	386
3_A	8,950,327	26,605	336	3_B	8,920,942	18,222	489
4_A	8,524,094	23,715	359	4_B	8,537,679	18,305	466
5_A	7,886,703	19,132	412	5_B	8,007,851	23,759	337
6_A	8,486,047	25,704	330	6_B	8,182,878	26,824	305
7_A	7,806,228	20,495	380	7_B	7,671,061	20,663	371
8_A	7,740,161	21,943	352	8_B	7,350,761	19,548	376
9_A	7,101,616	15,953	445	9_B	6,788,249	25,401	267
10_A	7,138,558	14,572	489	10_B	7,565,484	21,790	347
11_A	6,975,844	26,053	267	11_B	6,990,834	16,327	428
12_A	6,725,789	21,776	308	12_B	5,939,218	17,463	340
13_A	5,634,657	11,674	482	13_B	5,575,677	13,970	399
14_A	5,301,928	18,206	291	14_B	5,329,819	16,551	322
15_A	4,388,549	14,747	297	15_B	4,442,786	14,851	299
16_A	4,755,494	6,525	728	16_B	4,691,570	11,575	405
17_A	4,616,298	12,755	361	17_B	4,550,902	11,591	392
18_A	4,363,601	7,087	615	18_B	4,374,653	9,675	452
**Total**	**123,911,445**	**334,203**	**370**	**Total**	**122,076,697**	**328,470**	**371**

CD haplotype variants			
**Chromosome (20QLD87)**	**Length**	**Variants**	**Variants rate**
1_C	9,365,963	20,704	452
2_C	8,279,169	22,640	365
3_C	9,012,612	19,929	452
4_C	8,396,214	17,729	473
5_C	7,810,846	19,972	391
6_C	8,497,072	24,128	352
7_C	7,710,878	19,509	395
8_C	7,461,278	18,667	399
9_C	7,516,638	22,994	326
10_C	7,216,724	15,645	461
11_C	6,814,370	16,044	424
12_C	6,055,093	16,388	369
13_C	5,706,749	11,320	504
14_C	5,288,343	11,851	446
15_C	4,480,348	14,154	316
16_C	4,910,384	12,804	383
17_C	4,412,544	7,582	581
18_C	4,316,561	9,754	442
**Total**	**123,251,786**	**301,814**	**408**

Supplementary Table S7: Illumina read coverage, number of homozygous SNPs and *k*-mer containment score for the genomic loci of the b2, b3, b4 and b5 genes.

	Pt76 b2 (haplotype A)			*Pt76* b3 (haplotype B)			20QLD87 b4 (haplotype C)			20QLD87 b5 (haplotype D)			
	Coverage	# hom SNPs	k-mer score	Coverage	# hom SNPs	k-mer score	Coverage	# hom SNPs	k-mer score	Coverage	# hom SNPs	k-mer score	genotype
AU1 (AB)	**100%**	**0**	**100%**	**100%**	**0**	**100%**	70.8%	9	98.3%	83.4%	24	98.8%	b2/b3
AU2 (BC)	62.5%	26	97.9%	**100%**	**0**	**100%**	**100%**	**0**	**100%**	82.3%	34	98.8%	b3/b4
20QLD87 (CD)	62.4%	8	97.9%	79.6%	16	98.3%	**100%**	**0**	**100%**	**100%**	**0**	**100%**	b4/b5
AU5 (A)	**100%**	**0**	**100%**	67.5%	31	98.1%	66.8%	16	98.4%	75.3%	21	99.1%	b2/?
AU4	60.7%	8	98.2%	72%	15	98.5%	60.2%	16	98.5%	**100%**	**0**	**100%**	b5/?
09TUR23-1 (AB)	**100%**	**0**	**100%**	**100%**	**0**	**100%**	63%	6	99%	99.8%	8	99.8%	b2/b3
CZ10-9 (A)	**100%**	**0**	**100%**	74.9%	21	98.8%	64.8%	8	99%	94.8%	15	99.6%	b2/?
FR56 (A)	**100%**	**0**	**100%**	87.4%	19	99.2%	69.6%	12	98.9%	76.6%	15	99.3%	b2/?
EU1	74.6%	10	97.9%	74.3%	15	97.9%	56.7%	11	98.3%	96.4%	7	99.2%	?/?
EU4	**100%**	**0**	**100%**	**100%**	**0**	**100%**	62.9%	9	98.3%	75.5%	22	98.8%	b2/b3
EU5	91.8%	5	99.5%	74.7%	21	98.7%	76.2%	9	99.1%	94.6%	13	99.6%	?/?
NA1	98.2%	9	99.2%	71.1%	22	97.7%	64.9%	8	98.2%	75%	23	98.3%	?/?
NA2	89.2%	8	99.1%	72.2%	22	98%	67.8%	11	98.5%	81.3%	17	98.8%	?/?
NA3 (CD)	63.8%	8	98.1%	69.4%	16	98.3%	**99.9%**	**0**	**100%**	**99.9%**	**0**	**100%**	b4/b5
NA4 (D)	66.6%	7	98.7%	66.7%	18	98%	51.1%	6	98.6%	**100%**	**0**	**100%**	b5/?
NA5 (D)	70.6%	9	98.5%	74.1%	18	98.7%	69.5%	14	99%	**100%**	**0**	**100%**	b5/?
NA6 (D)	65.9%	11	98.2%	74.3%	20	98.6%	61.3%	19	98.7%	**100%**	**0**	**100%**	b5/?
NA7 (C)	72.2%	8	99%	64.6%	14	98.3%	99.9%	**0**	99.9%	79.9%	8	99.2%	b4/?
Durum	76.7%	14	98.2%	70.6%	16	98%	73.3%	10	98.4%	75.4%	20	98.5%	?/?
Middle East	**100%**	**0**	**100%**	64.1%	20	98.3%	**100%**	**0**	**100%**	61.4%	17	98.8%	b2/b4

Supplementary Table S8: Pathotyping results for the set of seven Australian isolates.

	Differential number	Resistance gene	Cultivar	19NSW04	19ACT06	19ACT07	19QLD08	20QLD87	20ACT90	20QLD91
International series		*Lr1*	Tarsa	V	A	V	A	V	V	V
		Lr2a	Webster	A	A	A	A	A	A	A
		Lr3a	Democrat	V	V	V	V	V	V	V
Australian series	1	*Lr20*	Thew	V	A	V	V	V	V	V
	2	*Lr23*	Gaza	A	A	A	A	A	A	A
	3	*Lr14a*	Spica	V	V	V	V	V	V	V
	4	*Lr15*	K1483	V	A	V	A	V	V	V
	5	*Lr3ka*	Klein Titan	V	V	V	V	A	V	V
	6	*Lr27+Lr31*	Gatcher	V	V	V	A	V	A	V
	7	*Lr17a*	Songlen	V	V	V	V	V	V	V
	8	*Lr28*	CS 2A/2M	A	A	A	A	V	A	A
	9	*Lr26*	Mildress	V	V	V	V	V	V	V
	10	*Lr13*	Egret	V	V	V	V	V	V	V
	11	*Lr16*	Exchange	A	A	A	A	A	A	A
	12	*Lr17b*	Harrier	V	V	V	V	V	V	V
	13	*Lr24*	Agent	A	V	A	V	A	A	A

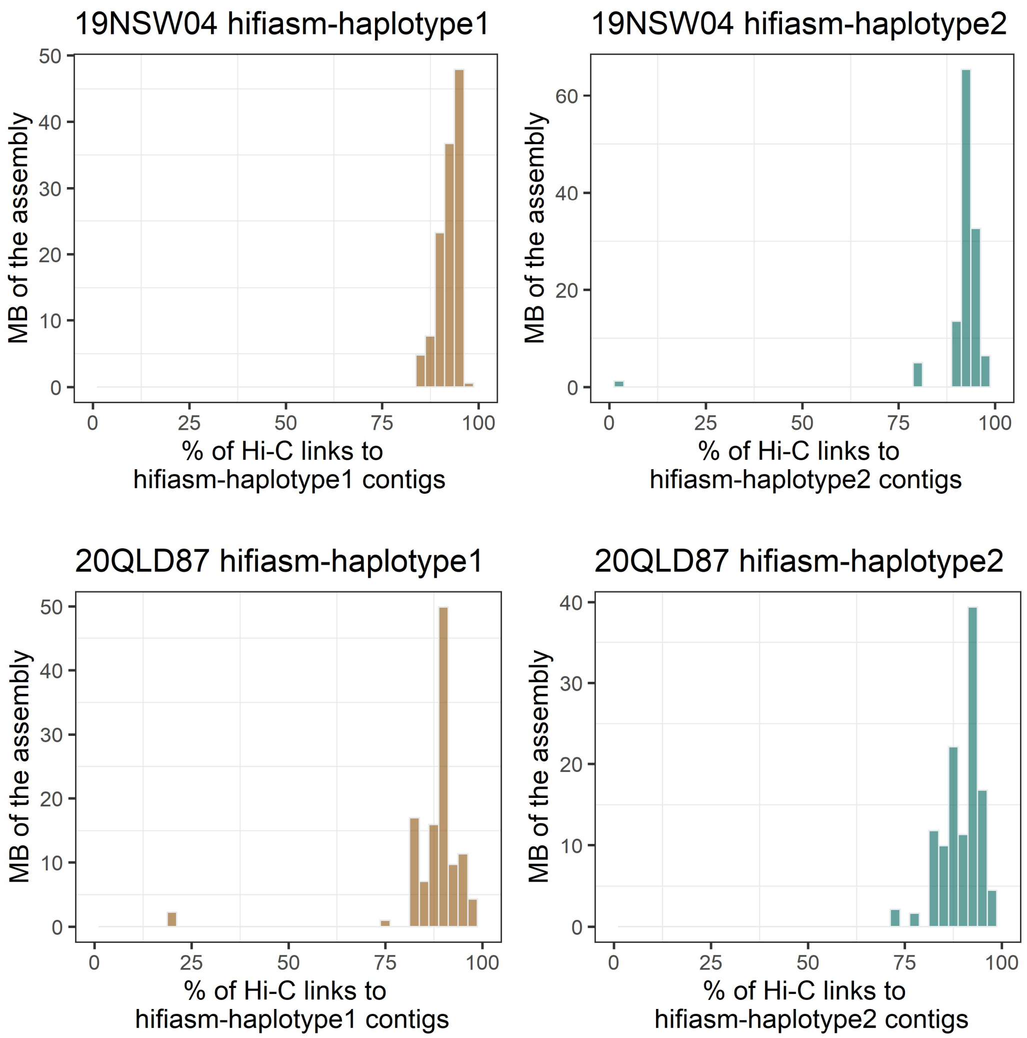

Figure S1: Hi-C *trans* contacts between the hifiasm-haplotype1 and hifiasm-haplotype2 in the 19NSW04 and 20QLD87 assemblies. Hi-C *trans* contacts for each contig were extracted from the HiC-Pro contact map in 100 Kb bins. The two assemblies exhibit a strong dikaryotic phasing signal. In both cases the haplotype1 and haplotype2 assemblies are close to perfectly nuclear-assigned, with only two contigs larger than 150 Kb (1.2 Mb in total) assigned to the incorrect phase in19NSW04 and only a single mis-assigned contig (2.2 Mb) in 20QLD87.

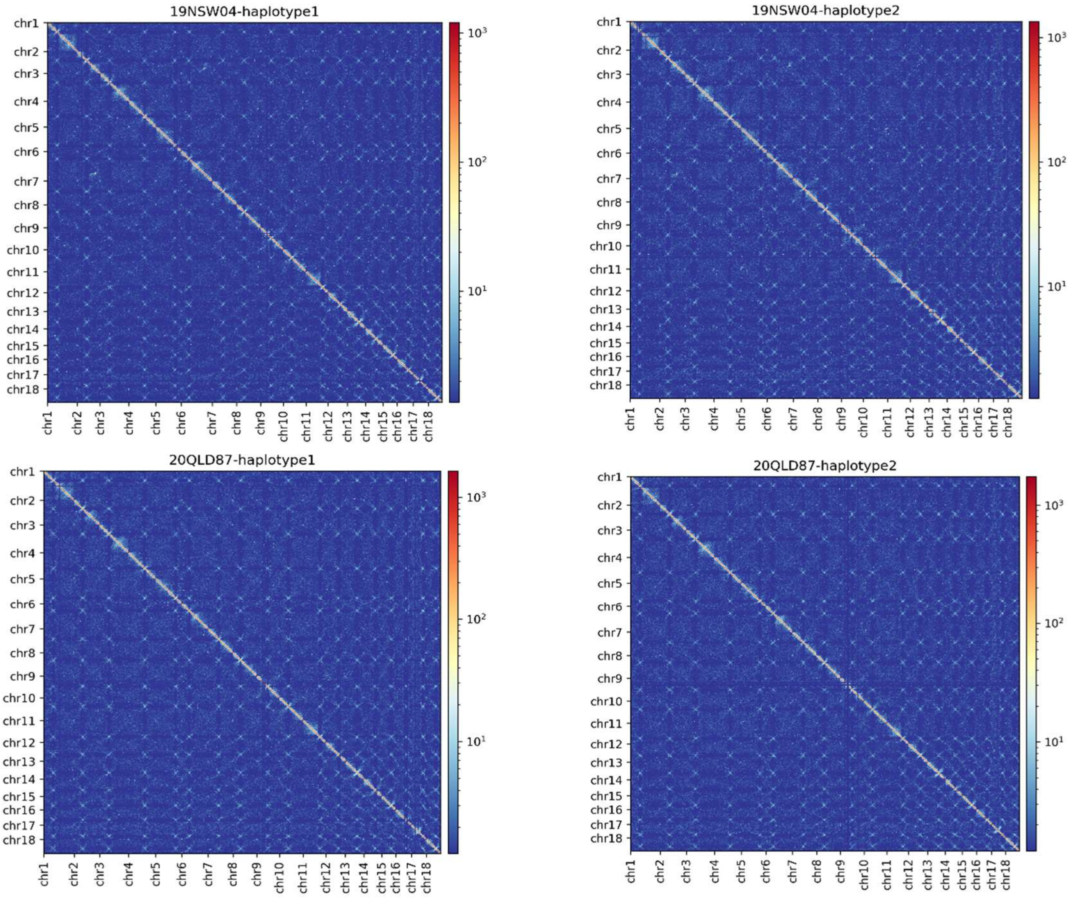

(A)

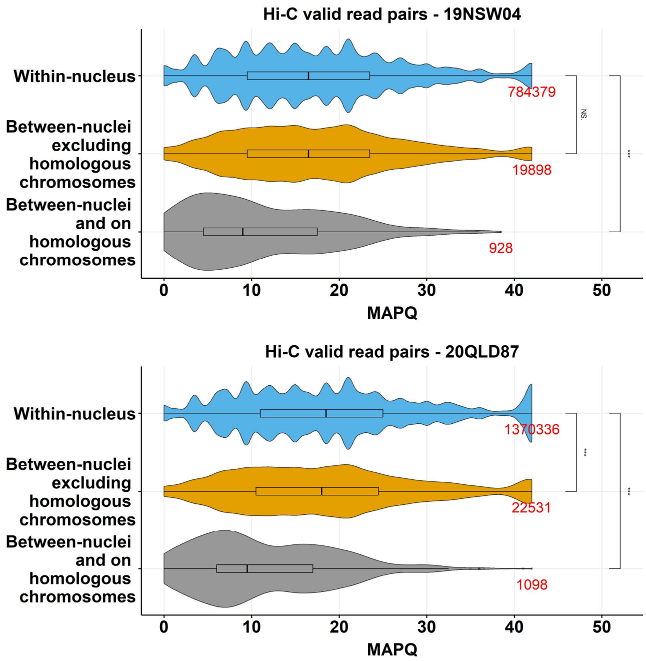

(B)

Figure S2: (A) Hi-C contact maps (100 Kb resolution) for the haplotype chromosomes of the two isolates 19NSW04 and 20QLD87. The 18 centromeres are visible as distinct outwards-spreading bowtie-like shapes. **(B) Mapping qualities (MAPQ) of Hi-C read pairs.** Total number of Hi-C read pairs are shown in red. Mapping quality (MAPQ) reflects the degree of confidence in the point of origin for a read. For example, MAPQ of 10 or less indicates that there is at least a 1 in 10 chance that the read originated from another genomic location. Mis-mapped Hi-C reads occur primarily between allelic, homologous chromosomes and as expected, these have significantly lower mapping quality in both isolates. In contrast, read pairs that occur between the two nuclei but are not on homologous chromosomes do not have significantly lower mapping in both isolates. Such Hi-C read pairs could be a result of some non-intact nuclei during the crosslinking. In the box plot, the boundaries represent the 25th percentile and the 75th percentile, with a line highlighting the median. The upper whisker extends from the hinge to the largest value no further than 1.5 *

interquartile range from the hinge. The lower whisker extends from the hinge to the smallest value at most 1.5 * interquartile range of the hinge. Outlier points are not shown in the box plot. *** indicates that the p-value from a t-test was < 0.001.

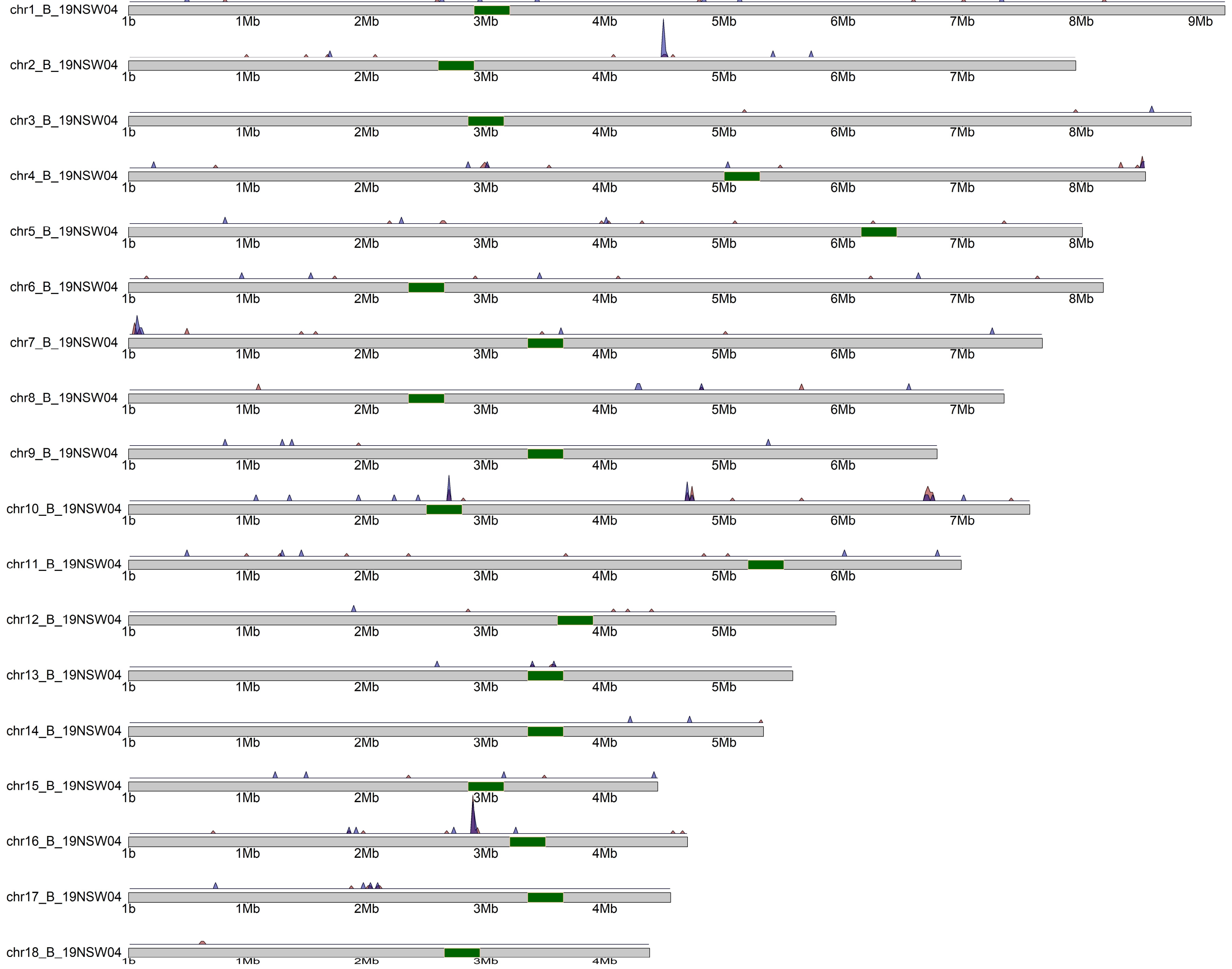

Figure S3: SNP density of the B haplotype variants in 20 KB windows. Non-synonymous SNPs are shown in red, synonymous SNPs are shown in blue.

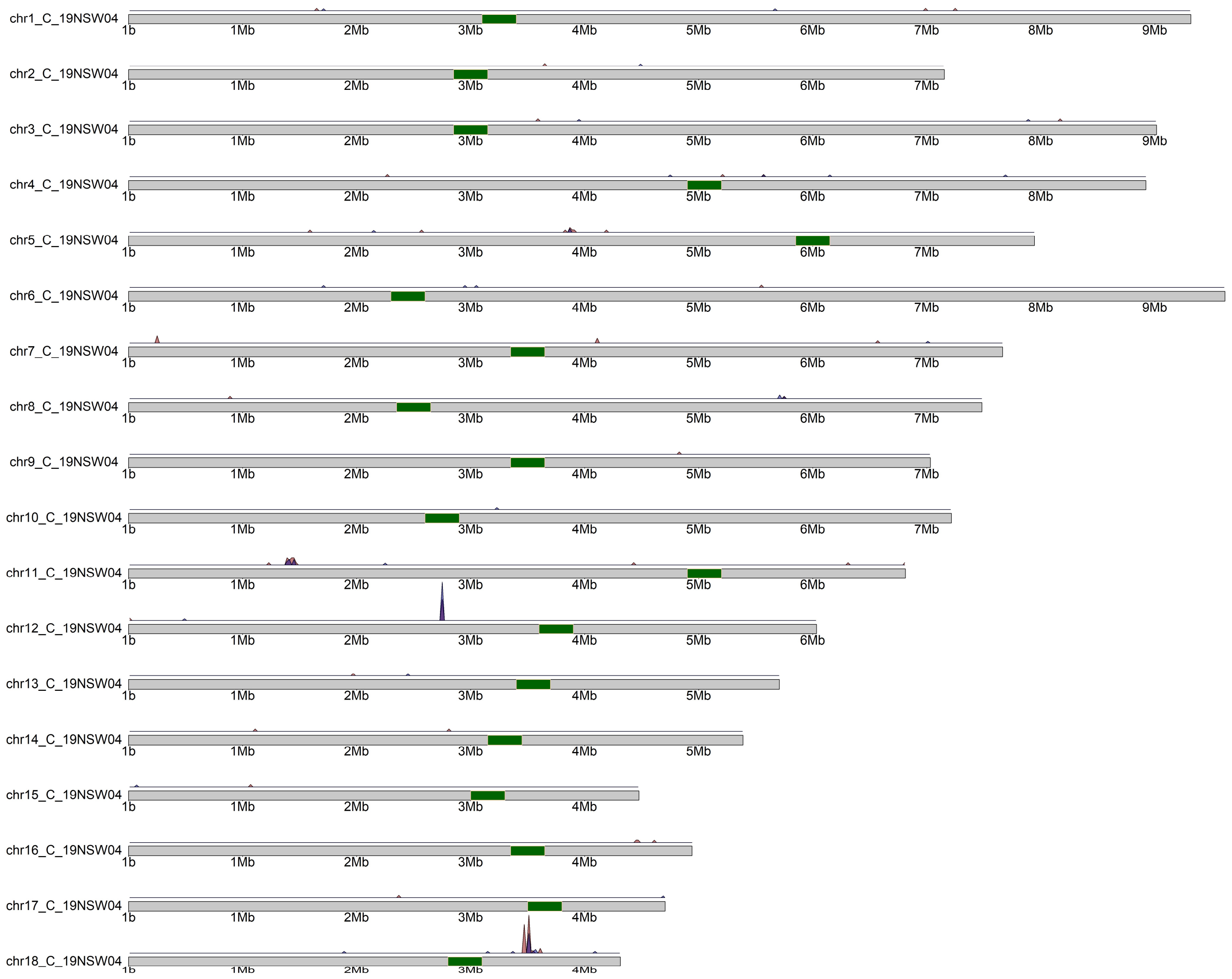

Figure S4: SNP density of the C haplotype variants in 20 KB windows. Non-synonymous SNPs are shown in red, synonymous SNPs are shown in blue.

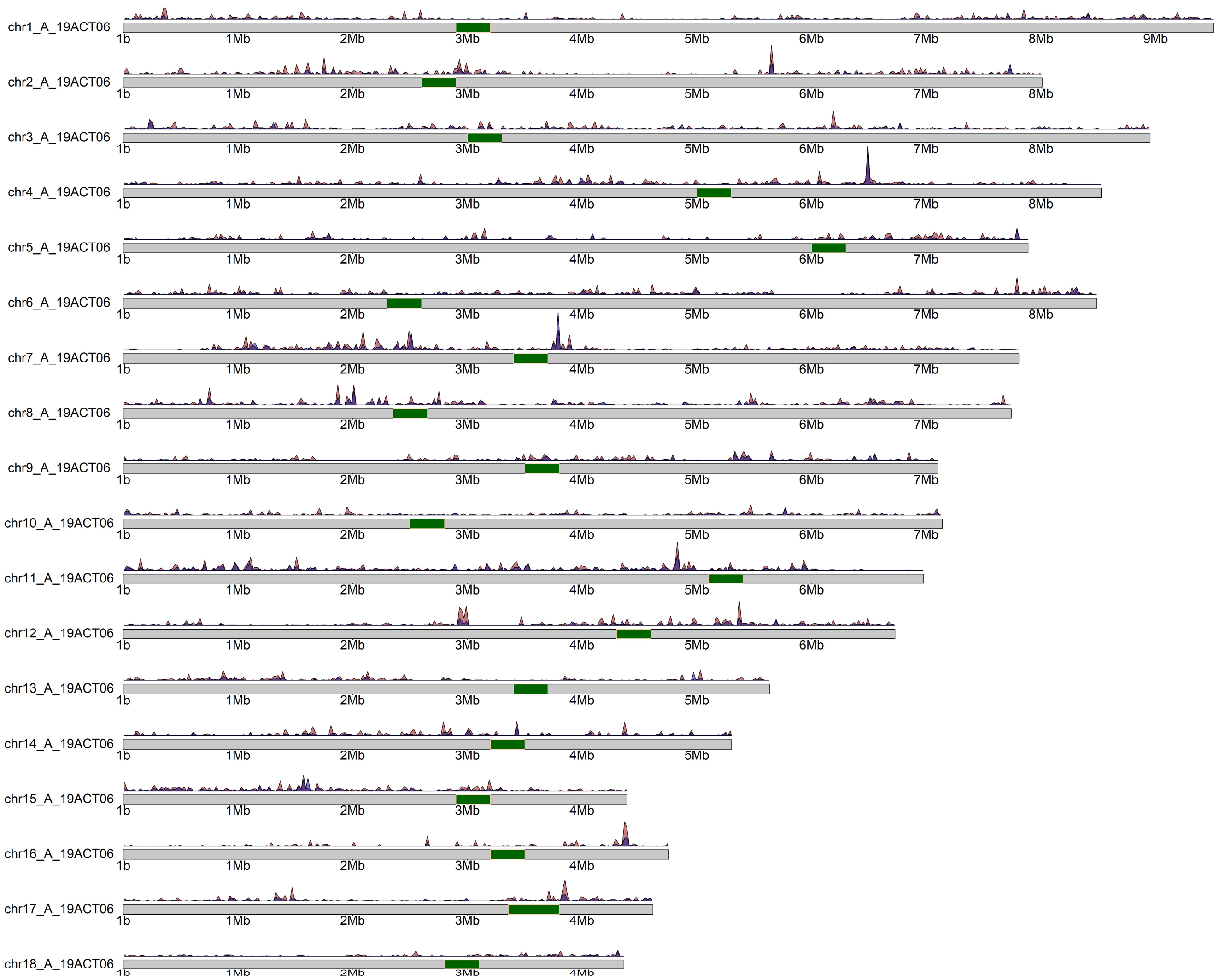

Figure S5: SNP density of the AB haplotype variants in 20 KB windows. Non-synonymous SNPs are shown in red, synonymous SNPs are shown in blue.

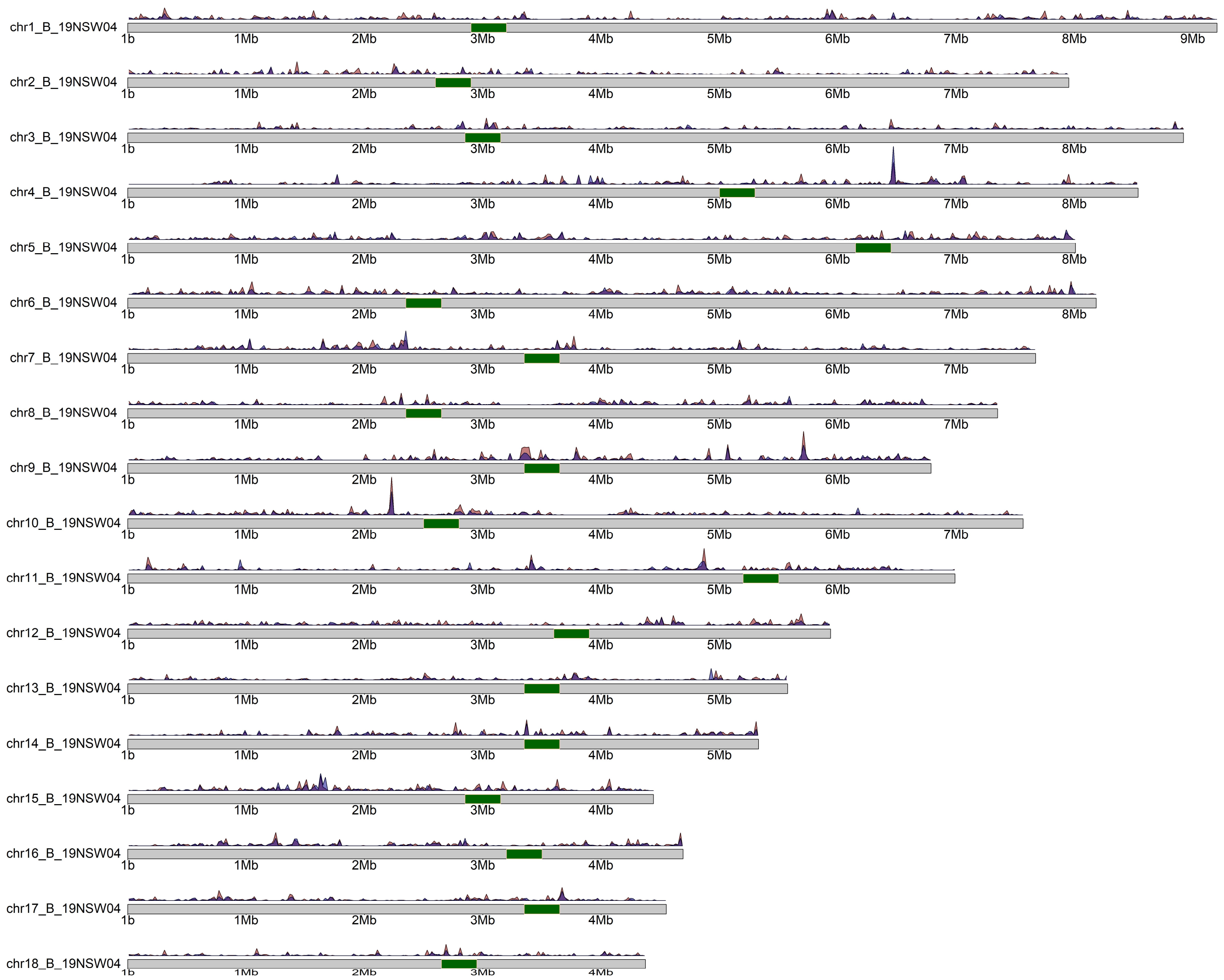

Figure S6: SNP density of the BC haplotype variants in 20 KB windows. Non-synonymous SNPs are shown in red, synonymous SNPs are shown in blue.

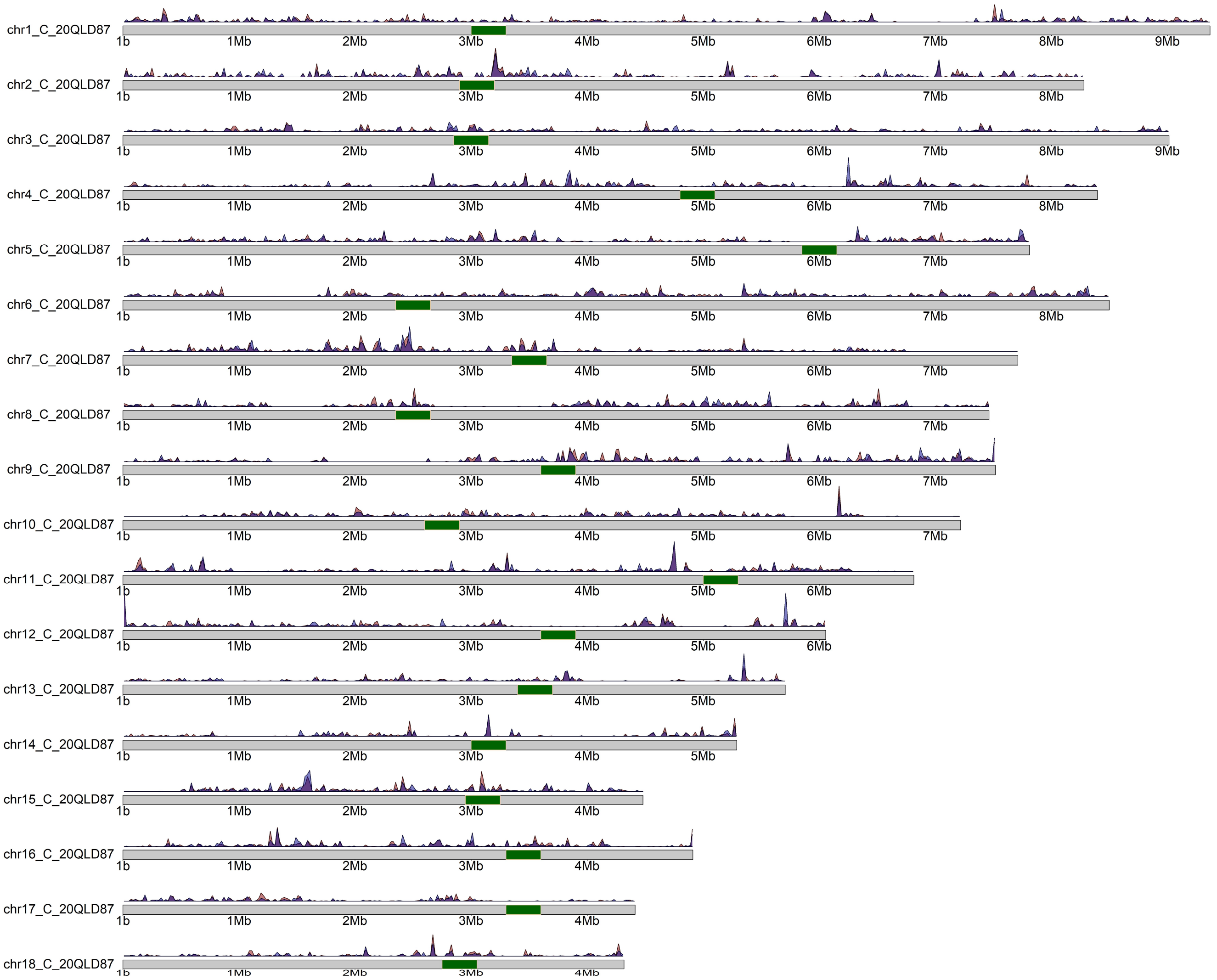

Figure S7: SNP density of the CD haplotype variants in 20 KB windows. Non-synonymous SNPs are shown in red, synonymous SNPs are shown in blue.

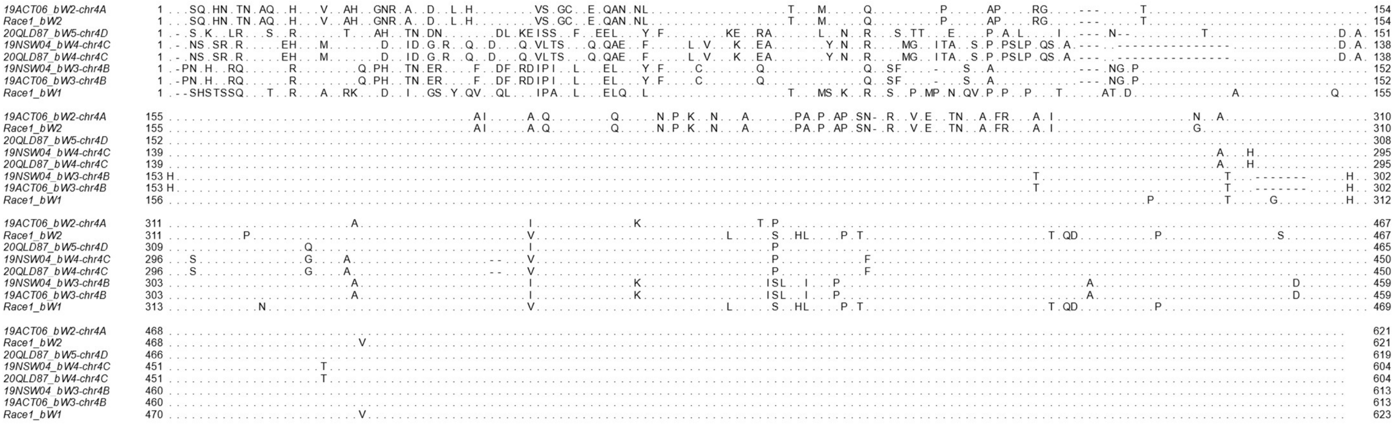

```
19NSW04_bE3-chr4B   1 - . . V . . . . A . . . . . . . . . . . SS . I . . A . L . D . . M . . . . . . - V . . . Q . . . . . S . . . . . . . V . I TP . N . . F . . . . . SC . LQ . . . . . . . . . . . . . . . . . R . . . AF . . . . . . . . . . . . . . . HR . . . . . . . . . . 127
19ACT06_bE3-chr4B   1 - . . V . . . . A . . . . . . . . . . . SS . I . . A . L . D . . M . . . . . . - V . . . Q . . . . . S . . . . . . . V . I TP . N . . F . . . . . SC . LQ . . . . . . . . . . . . . . . . . R . . . AF . . . . . . . . . . . . . . . HR . . . . . . . . . . 127
20QLD87_bE5-chr4D   1 - - MV . . . . A . S . P . . . . . S . AS . I . . A . M . D . . . . . R . . E - V . . . E . . . . . . . L . H . . N . . I TP . Q . . A . T . . . A . . L . . M . . . I . NT . . . . . . . . R . T . T . . . R . . L . . . . . W . . L . VV . . . . A . . I . 126
20QLD87_bE4-chr4C   1 - . I . SR . . . . . . . . . I . V . . . I . R . . . . AT . S . . LT . . . S . . I . . . . . . . . . . . . . . . . . Q . . . . S . . . . A . . . . . . . . . . . M . . . . . E . . . . . . . . . . . . DT . . . . . . . . I . . SQC . L . . V . . . . . . RM . 128
19NSW04_bE4-chr4C   1 - . I . SR . . . . . . . . . I . V . . . I . R . . . . AT . S . . LT . . . S . . I . . . . . . . . . . . . . . . . . Q . . . . S . . . . A . . . . . . . . . . . M . . . . . E . . . . . . . . . . . . DT . . . . . . . . I . . SQC . L . . V . . . . . . RM . 128
Race1_bE1           1 - . I I . N . . . . . TR . I . L . . . AA . L . . T . F . D . . N . . N . LH . I . L . Y . . . . . . . . . R . . Q . . . . Q . H . VL I HR . . . V . . . K . . . . . . E . F . . S . Q . . Y . . SEF . . . . . . . . . . . . . . . R . . . . . T . H . I . 128
Race1_bE2           1 . . . . . . . . . . L . S . . RAKT . V . R . . . . . . I . S . L . . RQRPV - L . . . . . . . . I . A . A . . . V . . . . . . Q . . . VL . DR . . . AA . . T . . . T . F . . FETA . R . . Y . KVE . . S . . P . . . . . . . . . . . . . . . . . . . I . . . W 128
19ACT06_bE2-chr4A   1 . . . . . . . . . . L . S . . RAKT . V . R . . . . . . I . S . L . . RQRPV - L . . . . . . . . I . A . A . . . V . . . . . . Q . . . VL . DR . . . AA . . T . . . T . F . . FETA . R . . Y . KVE . . S . . P . . . . . . . . . . . . . . . . . . . I . . . W 128


19NSW04_bE3-chr4B   128 . . . . . . . DI . L . . . . . . . . . . P . . . . . . . . . . . . . . . - . . . . . . . . . . SA . . . . . . . . . . . . . . . . . . . . . . . . . . . . . . . . . . . . . . . . Y . . K . . . . . . . . . . . . . . . . . . . . . . . . . . . . . . . . M . . . . . . . . . . 255
19ACT06_bE3-chr4B   128 . . . . . . . DI . L . . . . . . . . . . P . . . . . . . . . . . . . . . - . . . . . . . . . . SA . . . . . . . . . . . . . . . . . . . . . . . . . . . . . . . . . . . . . . . . Y . . K . . . . . . . . . . . . . . . . . . . . . . . . . . . . . . . . M . . . . . . . . . . 255
20QLD87_bE5-chr4D   127 . . . L . . . . A . . . . . . . . H . . . . . . . . . . . . . . . . . . . SS . . . . . . . . S . . . . . . . . . . . . . . . . . . . . . . . . . . . . . . . . . . . . . . . . . . . . . . . . . . . . . . . . . . . . . . . . . . . . . . . . . A . . . . . . . . V . . . . . 255
20QLD87_bE4-chr4C   129 VI . K . . . . . . T . . . T . . . . . . . . . . . . . . . . . . . . . . . - . . . . . . . . . . S . . . . . . . . . . . . . . . . . . . . . . . . . . . . . . . . . . . . . . . . . . . . . . . . . . . . . . . . . . . . . . . . . . . . . . . . . . . . . . . . . . . . . . 256
19NSW04_bE4-chr4C   129 VI . K . . . . . . T . . . T . . . . . . . . . . . . . . . . . . . . . . . - . . . . . . . . . . S . . . . . . . . . . . . . . . . . . . . . . . . . . . . . . . . . . . . . . . . . . . . . . . . . . . . . . . . . . . . . . . . . . . . . . . . . . . . . . . . . . . . . . 256
Race1_bE1           129 AL . . T . . . AFV . . . . . T . . . P . . . . . . . . . . . . . . . . - . . . . . . . . . . S . . . . . . . . . . . . . . . . . . . . . . . . . . . . . Y . . . D . E . . . . . . . L . . . . . . . . . . . . . . . . . . . . . . . . . . . . . . . . . . . . . . . . . . . . 256
Race1_bE2           129 . . . E . . AH . . . E . . . . . . . T . . . . . . . . P . . . . . . . - . . . . Y . H . . . A . . . . . . . . . . . . . . . . . . . . . . . . . . . . . Y . . . D . E . . . . . . . L . . . . . . . . . . . . . . . . . . . . . . . . . . . . . . . . . . . . . . . . . . . . 256
19ACT06_bE2-chr4A   129 . . . E . . AH . . . E . . . . . . . T . . . . . . . . P . . . . . . . - . . . . Y . H . . . A . . . . . . . . . . . . . . . . . . . . . . . . . . . . . Y . . . D . E . . . . . . . L . . . . . . . . . . . . . . . . . . . . . . . . . . . . . . . . . . . . . . . . . . . . 256


19NSW04_bE3-chr4B   256 . . . . . . . . . . . . . . . . . . . . . . . . . . . . . . . . . . . . . . . . . . . . . . . . . . . . . . . . . . . . . . . . . . . . . . . . . . . . . . . . . . . . . . . . . . . . . . . . . . . . . . . . . . . . . . . . . . . . . . . . . . . . . . . . . . . . . . . . . - - - -      373
19ACT06_bE3-chr4B   256 . . . . . . . . . . . . . . . . . . . . . . . . . . . . . . . . . . . . . . . . . . . . . . . . . . . . . . . . . . . . . . . . . . . . . . . . . . . . . . . . . . . . . . . . . . . . . . . . . . . . . . . . . . . . . . . . . . . . . . . . . . . . . . . . . . . . . . . . . - - - -      373
20QLD87_bE5-chr4D   256 . . . . . . . . . . . . . . . . . . . . . . . . . . . . . . . . . . . . . . . . . . . . . . . . . . . . . . . . . . . . . . . . . . . . . . . . . . . . . . . . . . . . . . . . . . . . . . . . . . . . . . . . . . . . . . . . . . . . . . . . . . . . . . . . . . . . . . . . . - - - -      373
20QLD87_bE4-chr4C   257 . . . . . . . . . . . . . . . . . . . . . . . . . . . . . . . . . . . . . . . . . . . . . . . . . . . . . . . . . . . . . . . . . . . . . . . . . . . . . . . . . . . . . . . . . . . . . . . . . . . . . . . . . . . . . . . . . . . . . . . . . . . . . . . . . . . . . . . . . - - - -      374
19NSW04_bE4-chr4C   257 . . . . . . . . . . . . . . . . . . . . . . . . . . . . . . . . . . . . . . . . . . . . . . . . . . . . . . . . . . . . . . . . . . . . . . . . . . . . . . . . . . . . . . . . . . . . . . . . . . . . . . . . . . . . . . . . . . . . . . . . . . . . . . . . . . . . . . . . L . . . .      378
Race1_bE1           257 . . . . . . . . . . . . . . . . . . . . . . . . . . . . . . . . . . . . . . . . . . . . . . . . . . . . . . . . . . . . . . . . . . . . . . . . . . . . . . . . . . . . . . . . . . . . . . . . . . . . . . . . . . . . . . . . . . . . . . . . . . . . . . . . . . . . . . . . . - - - -      374
Race1_bE2           257 . . . . . . . . . . . . . . . . . . . . . . . . . . . . . . . . . . . . . . . . . . . . . . . . . . . . . . . . . . . . . . . . . . . . . . . . . . . . . . . . . . . . . . . . . . . . . . . . . . . . . . . . . . . . . . . . . . . . . . . . . . . . . . . . . . . . . . . . . - - - -      374
19ACT06_bE2-chr4A   257 . . . . . . . . . . . . . . . . . . . . . . . . . . . . . . . . . . . . . . . . . . . . . . . . . . . . . . . . . . . . . . . . . . . . . . . . . . . . . D . I . . . . . . . . . . . . . . . . . . . . . . . . . . . . . . . . . . . . . . . . . . . . . . - - - -      374
```

Figure S8: Multiple sequence alignments of the bE and bW proteins.
