# Supplementary material for: Nuclear exchange generates population diversity in the wheat leaf rust pathogen Puccinia triticina
Source: Nat Microbiol. 2023 Oct 26;8(11):2130–41. doi: 10.1038/s41564-023-01494-9 (PMC10627818; doi:10.1038/s41564-023-01494-9)
Supplement: Supplementary file 2 — Reporting Summary [file 41564_2023_1494_MOESM2_ESM.pdf]

nature portfolio

Corresponding author(s): Jana Sperschneider, Peter Dodds, Melania Figueroa

Last updated by author(s): 27/8/23

Reporting Summary

Nature Portfolio wishes to improve the reproducibility of the work that we publish. This form provides structure for consistency and transparency in reporting. For further information on Nature Portfolio policies, see our Editorial Policies and the Editorial Policy Checklist.

Statistics

For all statistical analyses, confirm that the following items are present in the figure legend, table legend, main text, or Methods section.

n/a	Confirmed	
☐	☒	The exact sample size (*n*) for each experimental group/condition, given as a discrete number and unit of measurement
☒	☐	A statement on whether measurements were taken from distinct samples or whether the same sample was measured repeatedly
☐	☒	The statistical test(s) used AND whether they are one- or two-sided<br>*Only common tests should be described solely by name; describe more complex techniques in the Methods section.*
☒	☐	A description of all covariates tested
☒	☐	A description of any assumptions or corrections, such as tests of normality and adjustment for multiple comparisons
☐	☒	A full description of the statistical parameters including central tendency (e.g. means) or other basic estimates (e.g. regression coefficient) AND variation (e.g. standard deviation) or associated estimates of uncertainty (e.g. confidence intervals)
☐	☒	For null hypothesis testing, the test statistic (e.g. *F*, *t*, *r*) with confidence intervals, effect sizes, degrees of freedom and *P* value noted<br>*Give P values as exact values whenever suitable.*
☒	☐	For Bayesian analysis, information on the choice of priors and Markov chain Monte Carlo settings
☒	☐	For hierarchical and complex designs, identification of the appropriate level for tests and full reporting of outcomes
☒	☐	Estimates of effect sizes (e.g. Cohen's *d*, Pearson's *r*), indicating how they were calculated

Our web collection on statistics for biologists contains articles on many of the points above.

Software and code

Policy information about availability of computer code

Data collection	We used the following software as described in the Methods section:<br>hifiasm 0.16.1 , BLAST 2.11.0, minimap2 2.22, BUSCO 3.0.2, HiCanu 2.2.0, Merqury 1.3, BWA-MEM 0.7.17, bbmap, SALSA 2.2, HiC-Pro 3.1.0 , Hicexplorer 3.7.2, RepeatModeler 2.0.2a, RepeatMasker 4.1.2p1, HISAT2 2.1.0, Trinity 2.8.4, StringTie 2.1.6 , Funannotate 1.8.5, CodingQuarry 2.0, TransDecoder 5.5.0, SignalP 4.1, TMHMM 2.0, InterProScan 5.56-89.0, mummer 4.0.0rc1, SNPeff 5.1, bbmap, Mash 2.3, FreeBayes 1.3.5, trimmomatic v0.38, RAxML 8.2.12, mafft 7.4.90, iqtree2 2.2.0.8
Data analysis	Phasing of the assembled haplotypes was confirmed with the NuclearPhaser pipeline version 1.1 (https://github.com/JanaSperschneider/NuclearPhaser) (Duan et al., 2022).

For manuscripts utilizing custom algorithms or software that are central to the research but not yet described in published literature, software must be made available to editors and reviewers. We strongly encourage code deposition in a community repository (e.g. GitHub). See the Nature Portfolio guidelines for submitting code & software for further information.

Data

Policy information about availability of data

All manuscripts must include a data availability statement. This statement should provide the following information, where applicable:

- Accession codes, unique identifiers, or web links for publicly available datasets
- A description of any restrictions on data availability
- For clinical datasets or third party data, please ensure that the statement adheres to our policy

All sequence data and assemblies generated in this study are available at the NCBI BioProject PRJNA902835 (https://www.ncbi.nlm.nih.gov/bioproject/PRJNA902835). Sequencing reads, assemblies and gene annotation files are also available at the CSIRO Data Access Portal (https://data.csiro.au/collection/csiro:57097).

Human research participants

Policy information about studies involving human research participants and Sex and Gender in Research.

Reporting on sex and gender	N/A
Population characteristics	N/A
Recruitment	N/A
Ethics oversight	N/A

Note that full information on the approval of the study protocol must also be provided in the manuscript.

Field-specific reporting

Please select the one below that is the best fit for your research. If you are not sure, read the appropriate sections before making your selection.

☒ Life sciences ☐ Behavioural & social sciences ☐ Ecological, evolutionary & environmental sciences

For a reference copy of the document with all sections, see nature.com/documents/nr-reporting-summary-flat.pdf

Life sciences study design

All studies must disclose on these points even when the disclosure is negative.

Sample size	No sample size calculation was performed, instead we collected leaf rust samples from the field which represent the three prevalent lineages of leaf rust in Australia. We supplemented this with all publicly available sequencing data from different global regions.
Data exclusions	We checked all publicly available sequencing data for quality (coverage, allele frequencies). No data was excluded.
Replication	All computational analysis was successfully replicated multiple times, and code review was undertaken.
Randomization	For analysis of the sequencing data, randomization is not applicable.
Blinding	Not applicable.

Reporting for specific materials, systems and methods

We require information from authors about some types of materials, experimental systems and methods used in many studies. Here, indicate whether each material, system or method listed is relevant to your study. If you are not sure if a list item applies to your research, read the appropriate section before selecting a response.

Materials & experimental systems

n/a	Involved in the study	
☒	☐	Antibodies
☒	☐	Eukaryotic cell lines
☒	☐	Palaeontology and archaeology
☒	☐	Animals and other organisms
☒	☐	Clinical data
☒	☐	Dual use research of concern

Methods

n/a	Involved in the study	
☒	☐	ChIP-seq
☒	☐	Flow cytometry
☒	☐	MRI-based neuroimaging
